# Neat1 lncRNA organizes the inflammatory gene expressions in the dorsal root ganglion in neuropathic pain caused by nerve injury

**DOI:** 10.3389/fimmu.2023.1185322

**Published:** 2023-08-08

**Authors:** Motoyo Maruyama, Atsushi Sakai, Tsukasa Fukunaga, Yoshitaka Miyagawa, Takashi Okada, Michiaki Hamada, Hidenori Suzuki

**Affiliations:** ^1^ Department of Pharmacology, Nippon Medical School, Bunkyo-ku, Japan; ^2^ Division of Laboratory Animal Science, Nippon Medical School, Bunkyo-ku, Japan; ^3^ Waseda Institute for Advanced Study, Waseda University, Shinjuku-ku, Japan; ^4^ Department of Computer Science, Graduate School of Information Science and Technology, The University of Tokyo, Bunkyo-ku, Japan; ^5^ Department of Biochemistry and Molecular Biology, Nippon Medical School, Bunkyo-ku, Japan; ^6^ Division of Molecular and Medical Genetics, Center for Gene and Cell Therapy, The Institute of Medical Science, The University of Tokyo, Minato-ku, Japan; ^7^ Graduate School of Advanced Science and Engineering, Waseda University, Shinjuku-ku, Japan; ^8^ AIST-Waseda University Computational Bio Big-Data Open Innovation Laboratory (CBBD-OIL), Shinjuku-ku, Japan; ^9^ Graduate School of Medicine, Nippon Medical School, Bunkyo-ku, Japan

**Keywords:** dorsal root ganglion, neuropathic pain, long non-coding RNA, Neat1, microglia, neuroinflammation, cytokine

## Abstract

Primary sensory neurons regulate inflammatory processes in innervated regions through neuro-immune communication. However, how their immune-modulating functions are regulated in concert remains largely unknown. Here, we show that Neat1 long non-coding RNA (lncRNA) organizes the proinflammatory gene expressions in the dorsal root ganglion (DRG) in chronic intractable neuropathic pain in rats. Neat1 was abundantly expressed in the DRG and was upregulated after peripheral nerve injury. Neat1 overexpression in primary sensory neurons caused mechanical and thermal hypersensitivity, whereas its knockdown alleviated neuropathic pain. Bioinformatics analysis of comprehensive transcriptome changes indicated the inflammatory response was the most relevant function of genes upregulated through Neat1. Consistent with this, upregulation of proinflammatory genes in the DRG following nerve injury was suppressed by Neat1 knockdown. Expression changes of these proinflammatory genes were regulated through Neat1-mRNA interaction-dependent and -independent mechanisms. Notably, Neat1 increased proinflammatory genes by stabilizing its interacting mRNAs in neuropathic pain. Finally, Neat1 in primary sensory neurons contributed to spinal inflammatory processes that mediated peripheral neuropathic pain. These findings demonstrate that Neat1 lncRNA is a key regulator of neuro-immune communication in neuropathic pain.

## Introduction

Primary sensory neurons, whose cell bodies are located in the dorsal root ganglion (DRG), connect peripheral tissues to the spinal cord. Beyond their fundamental role of transmitting sensory information, primary sensory neurons are increasingly recognized as critically important factors in inflammation through neuro-immune communication in their innervated regions (1, 2). Accordingly, primary sensory neurons actively regulate peripheral immune responses in various inflammatory diseases and spinal neuroinflammation in pain disorders (3, 4). A variety of proinflammatory mediators induced in primary sensory neurons upon neuronal damage cooperatively activate spinal microglia, mediating chronic intractable neuropathic pain (5, 6). However, how inflammatory functions are organized in primary sensory neurons remains poorly understood.

Long non-coding RNAs (lncRNAs) are involved in a wide range of cellular processes and participate in the regulation of gene expression through various mechanisms (7). Many lncRNAs function as competing endogenous RNAs for microRNAs to indirectly regulate microRNA target genes or as antisense lncRNAs, which are transcribed from the opposite strand of the protein-coding gene, to regulate its host mRNA. In neuropathic pain, Kcna2 antisense lncRNA decreased the expression of its sense counterpart in primary sensory neurons (8). However, distinct from prevalent antisense lncRNAs, some lncRNAs have the potential to interact with non-host mRNAs, although little is known about the functional significance of the concurrent regulation of multiple specific targets.

Here, we identified Neat1 lncRNA as a critical regulator of the proinflammatory gene expressions in the DRG including primary sensory neurons in neuropathic pain. Neat1 is generally expressed in the nucleus to regulate gene expression (9) and has a profound role in pathological processes, such as cancer and inflammatory diseases (10, 11). We found that Neat1 was upregulated in the DRG to coordinate the expressions of inflammatory genes after peripheral nerve injury through RNA-RNA interaction-dependent and -independent mechanisms. Accordingly, Neat1 was causally involved in neuropathic pain and regulated spinal microglial activation through neuro-immune communication.

## Materials and methods

### Animal models

Male and female Sprague-Dawley rats (5–6 weeks of age) were used for all experiments. Food and water were provided *ad libitum*. All surgeries were performed on rats under deep anesthesia induced by the inhalation of isoflurane (2%–3%). To produce a neuropathic pain model, nerve injury was induced by lumbar fifth (L5) spinal nerve ligation (SNL) on the left (ipsilateral) side of rats, as previously described (12). The L5 spinal nerve was exposed and tightly ligated with 4-0 silk thread in two regions separated by about 1 mm. The inflammatory pain model was produced by the injection of 100 µl complete Freund’s adjuvant (CFA) (Sigma-Aldrich Japan, Tokyo, Japan) into the left plantar skin of the hind paw. The right (contralateral) side was left intact as a control.

### Behavioral tests

Behavioral tests (von Frey test and Plantar test) were performed, as previously described (13). A set of von Frey filaments (Muromachi Kikai, Tokyo, Japan) were used to measure paw withdrawal responses to mechanical stimuli. A von Frey monofilament was applied to the plantar surface of the hind paw of rat placed on a metallic mesh floor covered with a plastic box. The paw withdrawal threshold was referred to the smallest force required to induce withdrawal of the stimulated paw at least three times in five trials. Thermal hypersensitivity was examined using the Plantar Test (Ugo Basile, Varese, Italy). Each rat was placed on a glass plate with a radiant heat generator underneath. The average latency of paw withdrawal from the heat stimulus was measured twice separated by a 5-min interval and the mean was referred to as the paw withdrawal latency.

### RNA sequencing

The L5 dorsal root ganglion (DRG) was removed under deep anesthesia with isoflurane and total RNA was extracted using RNAiso plus (Takara Bio, Shiga, Japan). A polyadenylated RNA library was prepared using a TruSeq stranded mRNA LT sample Prep Kit (Illumina, San Diego, CA). Sequencing was performed on an Illumina HiSeq 2500 high-throughput sequencing system (75 bp pair-end directional reads). The reads were aligned to the Rattus norvegicus USCS rn5 reference genome with RefSeq gene annotation and were constructed into transcripts using RNA-Seq Alignment v1.0 (Illumina). FPKM values were calculated by Cufflinks Assembly & DE v2.0 (Illumina). Plausible lncRNA candidates of annotated genes were extracted from the gene database, HGCN (https://www.genenames.org/) and LNCipedia version 5.2 (https://lncipedia.org/). Differentially-expressed genes from the RNA-seq data were analyzed by Ingenuity Pathway Analysis (IPA, Qiagen K.K., Tokyo, Japan) to predict the functions of Neat1 in DRG neurons.

### Northern blotting

To detect full-length Neat1, an antisense RNA probe was produced. Fragments were amplified from the cDNA of rat DRG using forward (Neat1_1: 5′-CCAACACTGTGGGCTCTTGT-3′, Neat1_2: 5′-TCCCGTGACGAGTTTCCAAG-3′) and reverse primers (Neat1_1: 5′-TCCAATGTGACCAGCAAGCA-3′, Neat1_2: 5′-GGATGAGGGGCACACAGAAA-3′) attached with *Eco*RI and *Bam*HI restriction sites at 5′ ends, respectively, and were subcloned into the *Eco*RI and *Bam*HI sites of a pBluescriptII SK (–) plasmid vector (Agilent). After digestion of the plasmid with *Eco*RI, an antisense RNA probe conjugated with digoxigenin (DIG) was synthesized using T3 RNA polymerase with a DIG-labeling mix (Roche Diagnostics, Basel, Switzerland). For northern blotting, 10 µg of total RNA obtained from DRG was separated by electrophoresis on an RNA denaturing agarose gel (1% for Neat1_1 and 0.8% for Neat1_2), followed by blotting to a Hybond-N positively charged nylon membrane (GE Healthcare, Chicago, IL). After cross-linking at 203 nm UV light for 1 min in a transilluminator, the antisense RNA probe was hybridized in DIG Easy Hyb reagent (Roche Diagnostics) overnight at 68°C for Neat1_1 or at 72°C for Neat1_2. The membrane was washed in low stringency buffer (0.1% SDS and 2× SSC) at room temperature for 5 min, and then washed twice in high stringency buffer (0.1% SDS and 0.5× SSC) for 15 min at 68°C for Neat1_1 or at 72°C for Neat1_2. The membrane was incubated with 1% blocking solution (Roche Diagnostics) for 30 min, followed by a sheep anti-DIG antibody conjugated to alkaline phosphatase (1:10,000; catalog number 11093274910, Roche Diagnostics) for 30 min. A chemiluminescent reaction was performed using CDP-star (1:100; Roche Diagnostics) in detection buffer (100 mM Tris-HCl and 100 mM NaCl) and the signal was detected with a C-DiGit Blot Scanner (LI-COR Biotechnology, Lincoln, NE).

### Identification of the full-length rat Neat1 gene

Sequence homology between rat and mouse Neat1 sequences was analyzed using BLAST (https://blast.ncbi.nlm.nih.gov/). To obtain the complete cDNA sequence of the rat Neat1 gene, 5′ and 3′ RACE was performed using a GeneRacer Kit (Thermo Fisher Scientific, Waltham, MA) according to the manufacturer’s protocol. First-strand cDNA was synthesized from total RNA or polyadenylated RNA prepared using a Poly(A) Polymerase Tailing Kit (Cellscript, Madison, WI) by oligo (dT) or random hexamers using the SuperScript III First-Strand Synthesis System for RT-PCR (Thermo Fisher Scientific). Gene-specific primers for the RACE of rat Neat1 were designed for sequences homologous to mouse Neat1 (**Supplementary Table 1**). The 5′ and 3′ cDNA ends of Neat1_1 and Neat1_2 were amplified using Takara LA Taq DNA Polymerase (Takara Bio). PCR amplifications were performed at 94°C for 2 min as an initial denaturation step, followed by 30 cycles of amplification step (94°C for 30 sec, 57–68°C for 30 sec, and 72°C for 4 min). PCR products were subcloned into a pCRTM4-TOPO vector (Thermo Fisher Scientific) or pGEM-T Easy vector (Promega, Madison, WI) and subjected to Sanger sequencing using the BigDye Terminator v3.1 Cycle Sequencing kit (Thermo Fisher Scientific). Sequencing results were analyzed using Seq Scanner2 (Thermo Fisher Scientific).

### *In situ* hybridization

Rats were perfused transcardially with PBS (pH 7.2) followed by fresh 4% paraformaldehyde in PBS under deep anesthesia induced by isoflurane. The L5 DRG was excised and post-fixed in 4% paraformaldehyde overnight at 4°C, and then cryoprotected in 20% sucrose in PBS overnight at 4°C. Next, they were rapidly frozen in dry ice/acetone and sectioned (10 µm) using a cryostat (Leica Microsystems, Wetzlar, Germany). Tissues were pretreated with 1 µg/ml proteinase K (Merck, Darmstadt, Germany) at 37°C for 5 min and washed three times with PBS containing 0.1% Tween-20. Slides were incubated in 4% paraformaldehyde in PBS for 20 min, and then hybridized with the DIG-labeled RNA probe used in northern blotting in hybridization buffer (50% formamide, 5× SSC pH 4.5, 1% SDS, 50 µg/ml heparin sodium, and 50 µg/ml yeast RNA) at 65°C overnight. Slides were rinsed with first wash buffer (50% formamide, 5× SSC pH 4.5 and 1% SDS) at 65°C for 30 min and then three times with second wash buffer (50% formamide and 2× SSC pH 4.5) at 65°C for 30 min. Slides were incubated with 0.5% blocking solution (Roche Diagnostics) at room temperature for 1 h, and then with a sheep anti-DIG antibody (1:1000; catalog number 11333089001, Roche Diagnostics) at 4°C overnight. After slides were washed three times in TBS containing 0.1% Tween-20, they were incubated with an anti-sheep IgG antibody conjugated with Alexa Fluor 488 (1:1000; catalog number A11015, Thermo Fisher Scientific) at room temperature for 1 h. Fluorescent images were captured using a high-resolution microscope with a computer (Olympus, Tokyo, Japan).

### Quantitative PCR

All procedures were performed according to the corresponding manufacturers’ protocols. Total RNA was extracted from the L5 DRG using RNAiso plus (Takara Bio) and reverse-transcribed with a random primer using an iScript Select cDNA Synthesis Kit (Bio-Rad Laboratories, Hercules, CA). A PCR mixture was prepared using a Power SYBR Green PCR Master Mix (Thermo Fisher Scientific) or TaqMan Gene Expression Master Mix (Thermo Fisher Scientific) and premixed primer pairs or probes (**Supplementary Table 1**) specific for target genes. PCR amplifications were performed on a StepOnePlus Real-time PCR System (Thermo Fisher Scientific). The PCR program was initiated using 95°C for 10 min, followed by 40 cycles consisting of 95°C for 15 s and 60°C for 1 min for SYBR Green, and was initiated by 50°C for 2min and 95°C for 10 min, followed by 40 cycles consisting of 95°C for 15 s and 60°C for 1 min for TaqMan PCR. All samples were measured in triplicate. The relative expression was calculated according to the 2^-ΔΔCT^ method, as previously described (13).

### Viral vector production

Recombinant serotype 6 adeno-associated virus (AAV) vectors were produced by the adenovirus-free triple transfection method. The pAAV-GFP (14), AAV packaging (pRepCap 6as) (15), and helper (pHelper; Takara Bio) plasmids were co-transfected into HEK293EB cells at a ratio of 1:1:1 using polyethylenimine (Polysciences, Warrington, PA). Culture medium was collected at 5 days after transfection and cell debris was pelleted at 7000 rpm for 20 min at 4°C. AAV vectors were concentrated by ultrafiltration with hollow fiber 750 kDa and purified by cesium chloride density-gradient centrifugation gradient centrifugation at 30,000 rpm for 2.5 hours at 16°C. After dialysis with a Slide-A-Lyzer G2 dialysis cassette (Thermo Fisher Scientific), the AAV vector was concentrated using Amicon Ultra-4 30K (Merk Millipore, Burlington, MA). Genomic titers of each AAV vector were determined by quantitative PCR using EGFP-targeted forward (5′-GGCATCGACTTCAAGGAGGA-3′) and reverse primers (5′-TCGATGTTGTGGCGGATCTT-3′). For use, each AAV vector was diluted with PBS to approximately 5×10^13^ vector genomes (vg)/ml. AAV vectors (4 µl) were slowly injected into L5 DRG using a microsyringe with a 27-gauge needle after behavioral tests, as previously described (16).

The full-length Neat1_1 sequence was amplified using forward (5′-AGTGACAAGGAGGGC-3′) and reverse primers (5′-TCTCAAACCTTTATT-3′), which were attached at the 5′ ends with 15 bp of sequence homologous to the two ends of the linearized pAAV plasmid encoding an EGFP expression cassette produced by replacing the bGH pA sequence in the pAAV-mcs plasmid (Agilent Technologies, Santa Clara, CA) with a B19 promoter and EGFP gene. The Neat1_1 sequence was incorporated downstream of the CMV promoter in the linearized pAAV plasmid using an In-Fusion HD cloning kit (Takara Bio).

To knock down Neat1 expression, a short hairpin RNA (shRNA) for Neat1 and Neat1_2 was designed using Block-iT RNAi Designer (Thermo Fisher Scientific): Neat1 shRNA#1; 5′-GGGAATAATAGCTTGGGAACT-3′, shRNA#2; 5′-GGAGGTCGACTTTGAACTTGA-3′ and Neat1_2 shRNA; 5′-GGAAGGATCACACTGTCTTGA-3′. For the negative control, a scrambled shRNA sequence was designed using siRNA Wizard Software (InvivoGen, San Diego, CA): 5′-GGAGGTAGCATTGCGTAATAA-3′ for Neat1 control shRNA and 5′-GAACACGTGATCATGCTGTGA-3′ for Neat1_2 control shRNA. Top and bottom strands of the shRNA oligo were annealed and subcloned into a pSIH1-H1-copGFP shRNA vector (System Biosciences). This plasmid vector was digested further with *Acc*65I and the resultant fragment was subcloned into the *Acc*65I site of a pCAGS-EGFP&TNA plasmid vector (AAV expression plasmid).

### Immunofluorescence

*In vivo* transduction into DRG neurons was confirmed for all AAV vectors by immunofluorescence for EGFP. The DRG or spinal cord sections were pre-incubated in PBS containing 5% normal donkey serum and 0.2% Triton X-100 for 30 min, and then incubated with a rabbit anti-GFP antibody (1:1000; catalog number A11122, Thermo Fisher Scientific) or a rabbit anti-Iba1 antibody (1:250; Wako, Osaka, Japan) at 4°C overnight. After washing with PBS, the sections were incubated with a secondary antibody labeled with Alexa Fluor 488 (1:1000; catalog number A21206, Thermo Fisher Scientific) or Alexa Fluor 594 (1:1000; catalog number A21207, Thermo Fisher Scientific) at room temperature for 1 h. Images were captured using a high-resolution digital camera equipped with a computer (Olympus). Intensity of Iba1 immunofluorescence was quantified using Image J software (National Institutes of Health, Bethesda, MD).

### RIblast

Interaction energies between Neat1-regulated RNAs and Neat1 lncRNA were calculated using RIblast, an ultrafast RNA-RNA interaction prediction system based on the seed-and-extension approach (17). The potential target genes were sorted by energy of isoform that has a maximum length on each gene. RIblast calculates the interaction energy based on the intramolecular and intermolecular free energy between lncRNA and mRNA nucleotide sequences; thus, a lower energy indicates a higher interaction potential. We used −8000 kcal/mol as the interaction energy threshold. Because the sum of interaction energies for the whole Neat1 sequence for target RNAs were calculated, specific binding motifs such as seed regions of miRNA were not determined. The receiver operating characteristic (ROC) curve analysis for RNA-RNA interaction prediction was performed using pROC R package.

### RNA pull-down

RNA pull-down was performed according to previous reports with minor modifications (18, 19). Briefly, rat DRGs were fixed with 1 ml of 1% paraformaldehyde solution for 10 min and homogenized in 500 µl of proteinase K solution (100 mM NaCl, 10 mM Tris-HCl, 0.1 mM EDTA, 0.5% SDS, and 200 U/ml RNase OUT). Lysates were incubated with proteinase K (20 mg/ml; Thermo Fisher Scientific) for 45 min at 50°C followed by 13 min at 95°C. One milliliter of hybridization buffer (700 mM NaCl, 70 mM Tris-HCl, 0.1 mM EDTA, 1.25% SDS, and 200 U/ml RNase OUT and 15% formamide) was added and 20 µl of samples were collected as input samples. Neat1-specific (5′-GCCTTCCCACATTTAAAAACACAAC-3′) or negative control (5′-TAAAATACCATTTGATGTTTGAAATTAT-3′) oligonucleotide probes (18) biotinylated at the 3′-end (100 pmol) were added and incubated for 4 h at room temperature with agitation in a rotator. Magnetic beads (Dynabeads MyOne Streptavidin C1, Thermo Fisher Scientific) were added and incubated at room temperature overnight with agitation. Beads were washed in wash buffer (2× SSC and 0.5% SDS) five times. Beads were incubated with proteinase K for 45 min at 50°C followed by 10 min at 95°C. RNA was purified with RNA Clean & Concentrator-5 (Zymo Research, Irvine, CA) and genomic DNA was digested by DNase for 30 min at 37°C. Eluted RNA was reverse transcribed using SuperScript IV reverse transcriptase (Thermo Fisher Scientific) and was subject to qPCR for the detection of enriched transcripts.

### RNA-decay assay

The L4–L6 DRGs were removed under deep anesthesia with isoflurane. DRGs were digested with 5 mg/ml collagenase A (Roche Diagnostics) and 1 mg/ml dispase II (Roche Diagnostics) for 30 min at 37°C, followed by 0.05% Trypsin/EDTA (Wako) for 30 min at 37°C. Then, DRGs were dissociated in F-12 medium (Thermo Fisher Scientific) supplemented with 15% fetal bovine serum by gentle pipetting. Cells were washed with F-12 medium and were resuspended in Neurobasal medium (Thermo Fisher Scientific) supplemented with 1% B27 supplement (Thermo Fisher Scientific) and 1% GlutaMAX (Thermo Fisher Scientific). Cells were plated onto 48-well plate coated with 0.5 mg/ml poly-L-lysine (Nacalai tesque, Kyoto, Japan) and 10 µg/ml Laminin I (R&D systems, Minneapolis, MN). Control or Neat1 AAV vector was added at 5×10^9^ vector genomes (vg)/well. Forty-eight hours after transduction, the cells were treated with 5 µg/ml actinomycin D (Wako) or 100 µM 5,6-dichlorobenzimidazole 1-β-D-ribofuranoside (DRB; Sigma-Aldrich Japan) to block the transcription and were collected 0, 4, 6, and 8 h after actinomycin D treatment or 8 h after DRB treatment. Quantification of mRNA levels were performed as described in the quantitative PCR method.

### Cytokine administration

A polyethylene catheter (PE-10) filled with saline was inserted into the subarachnoid space between cranial bone and atlas before AAV injection of the L5 DRG. The tip of the catheter was inserted to the level of lumbar spinal cord (20). Rats with hindlimb paralysis were excluded from the experiments. Cytokine cocktails (100 ng each of CCL2, CCL7, CXCL9, IL-1β, and LIF; PeproTech, Cranbury, NJ) in 10 µl saline followed by 10 µl of saline (flush) was administered 14 days after SNL surgery through the intrathecal catheter.

### Enzyme linked immunosorbent assay (ELISA)

The L5 dorsal spinal cord was homogenized with Cell Disruption Buffer in mirVana PARIS RNA and Native Protein Purification Kit (Thermo Fisher Scientific). Lysates were centrifuged at 12,000 rpm for 20 min at 4°C and the supernatants were collected. ELISA was performed using RayBio ELISA kits for rat CCL2, IL-1β, and IL-6 (RayBiotech, Norcross, GA) according to the manufacturer’s protocols.

### Statistics

Values are expressed as the mean ± standard error of the mean (SEM). SPSS software (IBM, Armonk, NY) was used for statistical analyses. Normality of data was assessed by the Shapiro-Wilk test. The paired *t*-test and unpaired *t*-test were used for normally distributed data sets. When normality was rejected, the Mann–Whitney *U*-test was used. The two-way ANOVA was used to assess differences between the effects of SNL and AAV treatment, followed by a *post hoc* Tukey’s test. *P* < 0.05 was considered statistically significant.

## Results

### Neat1 lncRNA is upregulated in the nuclei of DRG cells after nerve injury

**Figure 1:**
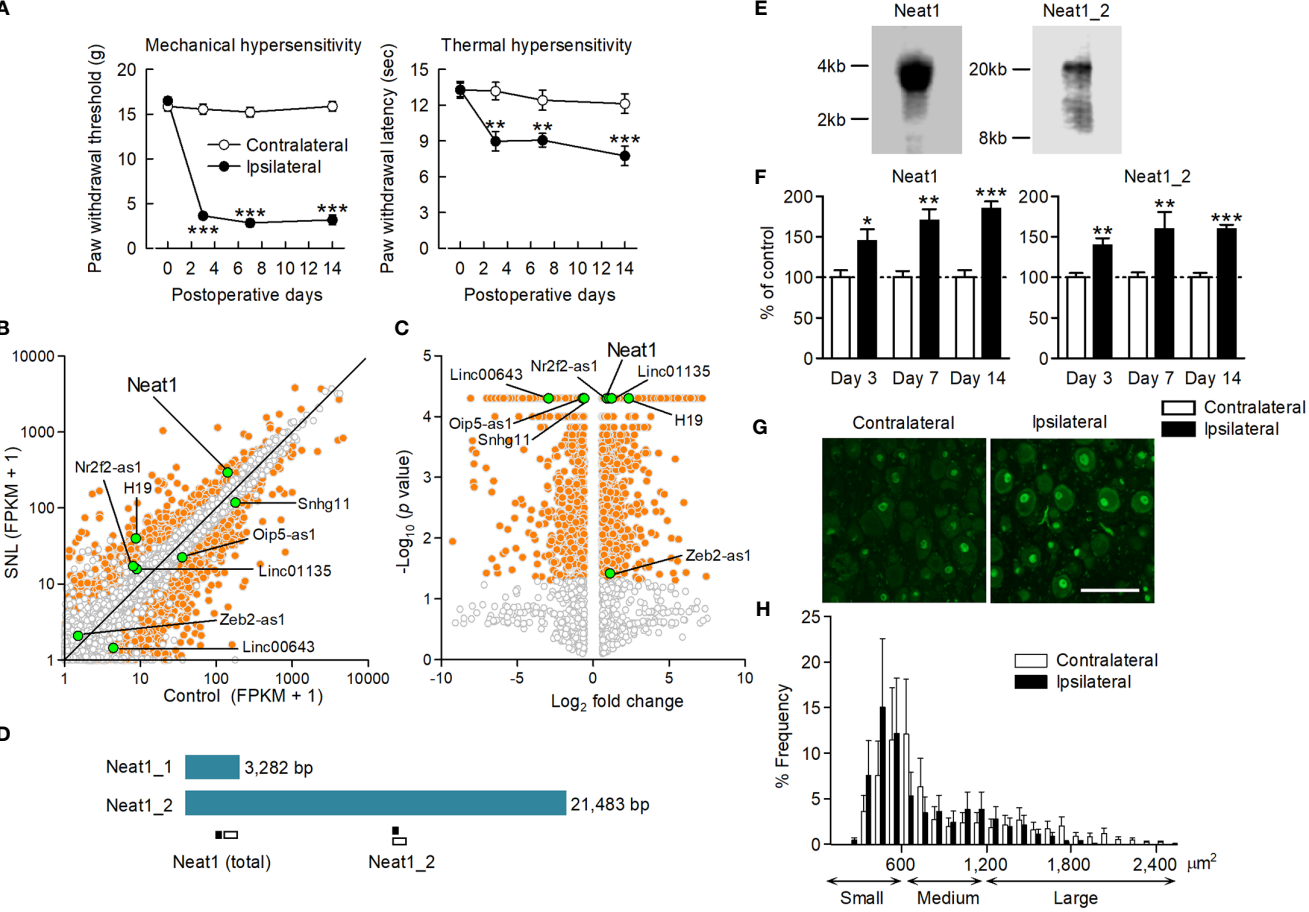
Neat1 lncRNA upregulation in the DRG after nerve injury. **(A)** Paw withdrawal thresholds and latencies to mechanical and thermal stimuli, respectively, were evaluated on the ipsilateral and contralateral sides. **P < 0.01 and ***P < 0.001 compared with the contralateral side, paired *t*-test (*n* = 11). **(B, C)** Transcriptome changes were examined in the L5 DRG 14 days after SNL using RNA sequencing. The orange and white dots represent genes with significant and non-significant expression changes, respectively (fold change > 1.5 or < 0.67, *P* < 0.05, *n* = 3). The green dots represent lncRNAs, including lncRNA candidates, that showed significant expression changes. **(D)** Schematic representation of Neat1 isoforms. The positions of the primer used for qPCR (black bar) and the probe for *in situ* hybridization (white bar) were shown. **(E)** The full length of Neat1 expression in the DRG was shown by northern blotting. **(F)** The time course of expression changes of Neat1 and Neat1_2 was examined in the L5 DRG after SNL using qPCR. *P < 0.05, **P < 0.01, and ***P < 0.001 compared with the contralateral side, Mann-Whitney *U*-test (*n* = 8–9). **(G, H)** Representative images **(G)** and size distribution **(H)** of Neat1 expression in the L5 DRG 14 days after SNL (*n* = 4). Scale bar, 100 µm.

To explore potential lncRNAs responsible for neuropathic pain, we performed a comprehensive analysis of gene expression changes in the L5 DRG where the cell bodies of primary sensory neurons are located. SNL was performed on rats to produce a neuropathic pain model (12). After SNL, the paw withdrawal threshold and latency were significantly decreased in response to mechanical and thermal stimuli, respectively (**Figure 1A**), indicating the occurrence of neuropathic pain. RNA sequencing revealed that many genes, including protein-coding genes, annotated in the RefSeq rat genome database (Rnor_5.0) as well as non-annotated potential genes, were differentially expressed after SNL (**Figures 1B, C**). Annotated genes contained only a few differentially-expressed lncRNAs because of the poor registration of lncRNAs in the rat gene database (**Supplementary Table 2**). However, we found several more lncRNA candidates with altered expression from the non-annotated potential genes based on sequence homology with mouse and human lncRNAs using the UCSC genome browser (https://genome.ucsc.edu/) and BLAST (https://blast.ncbi.nlm.nih.gov/) (**Supplementary Table 3**). Of these, the lncRNA candidate with the highest expression level after nerve injury (FPKM: CNT_140.56, SNL_293.65; fold change: 2.09; *P* < 0.001; **Figures 1B, C** and **Supplementary Tables 2**, **3**) was highly homologous to mouse Neat1 lncRNA. Therefore, we determined the nucleotide sequence of rat Neat1 lncRNA in the DRG by the 5′ and 3′ Rapid Amplification of cDNA Ends. Short and long isoforms of Neat1, which share the same 5′ end, were located in chromosome 1:221201000-221204281 (3,282 bp) and chr1:221182799-221204281 (21,483 bp) in RGSC 5.0/rn5, respectively (**Figure 1D**). Consistent with the results of RACE, northern blotting revealed the bands of Neat1_1 and Neat1_2 lncRNA were approximately 3 kb and >20 kb, respectively (**Figure 1E**). Neat1 (sum of Neat1_1 and Neat1_2) was highly expressed in the nervous systems including DRG, spinal cord and brain (**Supplementary Figure 1**), although a previous report indicated that its expression was higher in stomach and intestine in mice (21).

Neat1 and Neat1_2 were significantly increased in the L5 DRG from day 3 to 14 after SNL (**Figure 1F**). In contrast, the expression levels of Neat1 and Neat1_2 were not changed in the injury-spared L4 DRG and L5 dorsal spinal cord after SNL (**Supplementary Figure 2**). Furthermore, their expressions were unchanged in the L5 DRG during inflammatory pain (**Supplementary Figure 3**), suggesting that Neat1 was preferentially upregulated after nerve injury. *In situ* hybridization revealed that Neat1 and Neat1_2 were expressed in DRG cells; mainly localized in the nucleus of DRG neurons, but also in non-neuronal cells (**Figure 1G** and **Supplementary Figure 4A**). Both isoforms were broadly expressed in small cell-sized neurons, which are putative C-fiber neurons, and medium-to-large cell-sized neurons, which are putative A-fiber neurons (**Figure 1H** and **Supplementary Figure 4B**). The intracellular distribution and frequency of positive cells containing Neat1 isoforms were not affected by nerve injury.

### Neat1 in primary sensory neurons contributes to neuropathic pain

**Figure 2:**
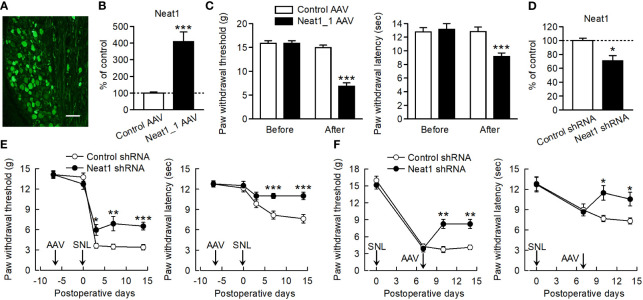
Neat1 in primary sensory neurons contributes to neuropathic pain. **(A)** Representative image of EGFP immunofluorescence in the L5 DRG 7 days after injection of the AAV vector encoding Neat1_1 and EGFP. Scale bar, 100 µm. **(B, D)** Expressions of Neat1 in the L5 DRG 14 days after control or Neat1_1 AAV injection **(B)** and 14 days after SNL in rats injected with control or Neat1 shRNA AAV **(D)**. *P < 0.05 and ***P < 0.001 compared with the control AAV and control shRNA AAV, Mann-Whitney *U*-test [**(B)** *n* = 8 and **(D)** *n* = 9]. **(C, E, F)** Paw withdraw thresholds and latencies to mechanical and thermal stimuli, respectively, were evaluated before and 14 days after control or Neat1_1 AAV injection **(C)** and control or Neat1 shRNA AAV injection 7 days before **(E)** and after **(F)** SNL. *P < 0.05, **P < 0.01, and ***P < 0.001 compared with control AAV injection to intact rats [**(C)**; *n* = 11] and control shRNA injection to SNL rats [**(E)**; n = 12 and **(F)**; n = 6–7], Mann-Whitney *U*-test for mechanical hypersensitivity, unpaired *t*-test for thermal hypersensitivity.

To examine the involvement of Neat1 in nociceptive processing, Neat1_1 was overexpressed specifically in primary sensory neurons of intact rats using a serotype 6 AAV vector. Injection of the AAV vector induced EGFP expression in the L5 DRG neurons of all cell sizes (**Figure 2A** and **Supplementary Figure 5**), as described previously (22). Neat1_1 overexpression caused pronounced mechanical and thermal hypersensitivity (**Figures 2B, C**). Then, we investigated the involvement of Neat1 in neuropathic pain by suppressing Neat1 expression specifically in the injured L5 DRG neurons. An AAV vector encoding shRNA for Neat1 significantly decreased the expression of Neat1 after nerve injury (**Figure 2D** and **Supplementary Figure 6A**). Preemptive Neat1 knockdown in primary sensory neurons reduced mechanical and thermal hypersensitivity following nerve injury (**Figure 2E** and **Supplementary Figure 6B**). Furthermore, Neat1 knockdown also alleviated neuropathic pain after the pain had developed (**Figure 2F**). To examine the involvement of Neat1_2 in neuropathic pain, Neat1_2 expression in primary sensory neurons was downregulated using a Neat1_2 shRNA (**Supplementary Figure 7A**). Neat1_2 knockdown also reduced mechanical and thermal hypersensitivity after nerve injury (**Supplementary Figure 7B**), suggesting that Neat1 contributes to the maintenance of neuropathic pain.

### Neat1 regulates the proinflammatory function in the DRG following nerve injury

**Figure 3:**
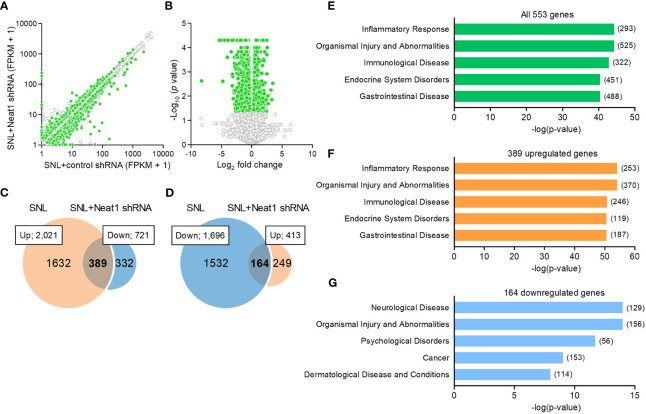
Neat1-regulated genes are associated with inflammatory response and neurological disease in neuropathic pain. **(A, B)** Transcriptome changes were examined in the L5 DRG 14 days after SNL in rats injected with the AAV vector encoding control or Neat1 shRNA 7 days before SNL using RNA sequencing. The green and white dots represent genes with significant and non-significant expression changes, respectively (*P* < 0.05, *n* = 3). **(C, D)** Venn diagram of genes upregulated by SNL and genes downregulated by Neat1 shRNA **(C)**, and genes downregulated by SNL and genes upregulated by Neat1 shRNA **(D)**. **(E–G)** Top five of diseases and disorders associated with all 553 genes **(E)**, 389 upregulated genes **(F)** and 164 downregulated genes **(G)** regulated by Neat1 in neuropathic pain were assessed by IPA. The number of genes involved in the pathways was shown in brackets.

Because Neat1 was preferentially expressed in the nucleus of neurons, we explored the impact of Neat1 inhibition on transcriptomic changes in the DRG after nerve injury using RNA sequencing (**Figures 3A, B**). In SNL rats, Neat1 knockdown decreased 721 annotated genes (**Figure 3C**) and increased 413 annotated genes (**Figure 3D**). Among 2,021 annotated genes upregulated by SNL (**Figures 1B, C**), the expressions of 389 genes (19.25%) were suppressed by Neat1 knockdown (**Figure 3C** and **Supplementary Table 4**). On the other hand, among 1,696 annotated genes downregulated by SNL (**Figures 1B, C**), the expressions of 164 genes (9.67%) were significantly upregulated by Neat1 knockdown (**Figure 3D** and **Supplementary Table 5**). Next, we explored the functions associated with these Neat1-regulated genes using bioinformatic analysis. IPA revealed that the most relevant diseases and disorders for 553 genes (389 + 164 genes) whose expression changes after SNL were inhibited by Neat1 knockdown was the inflammatory response (**Figure 3E**). The inflammatory response was also identified when limited to 389 genes upregulated through Neat1 (**Figure 3F**), whereas the most relevant diseases and disorders for 164 genes downregulated through Neat1 was the neurological disease (**Figure 3G**).

**Figure 4:**
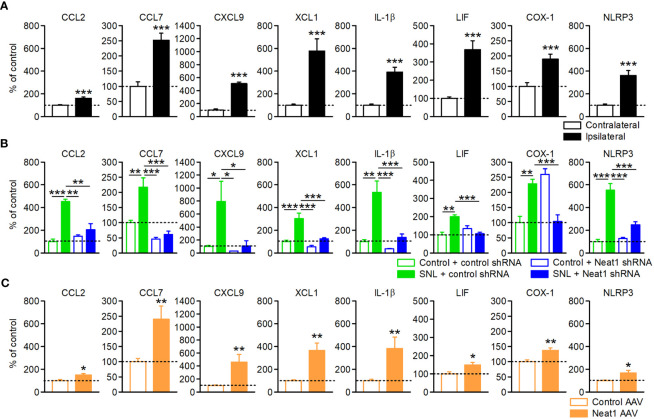
Neat1 coordinates the expressions of diverse inflammatory genes in the DRG. Expressions of inflammatory genes in the L5 DRG 14 days after SNL **(A)**, 14 days after SNL in rats injected with the AAV vector encoding control or Neat1 shRNA, as a percentage of the expression level in the L5 DRG of intact rats injected with control shRNA AAV vector **(B)**, and 14 days after control or Neat1_1 AAV injection were examined using qPCR **(C)**. *P < 0.05, **P < 0.01, and ***P < 0.001 compared with the contralateral side [**(A)**; *n* = 10], and the control AAV injection to intact rats [**(C)**; *n* = 9–10], Mann-Whitney *U*-test. *P < 0.05, **P < 0.01, and ***P < 0.001, two-way ANOVA followed by Tukey’s test [**(B)**; *n* = 8–12].

Bioinformatics analysis implicated the significance of inflammatory regulation by Neat1 in neuropathic pain. Of note, we observed that the inflammatory genes (**Supplementary Table 4**) included multiple factors involved in the spinal microglial activation, which is critically involved in the maintenance of neuropathic pain (5). These included genes encoding chemokines [CCL2 (23, 24), CCL7 (24, 25), CXCL9 (26, 27), and XCL1 (28, 29)], interleukin family cytokines [IL-1β (30, 31) and LIF (32, 33)], and others [COX-1 (34, 35) and NLRP3 (36, 37)]. qPCR confirmed that the inflammatory genes were increased in the L5 DRG after SNL (**Figure 4A**), and that these upregulations were suppressed by Neat1 knockdown (**Figure 4B**), consistent with the RNA sequencing results (**Supplementary Figure 8**). On the other hand, knockdown of Neat1_2 alone suppressed the expressions of only CCL2 and CCL7 (**Supplementary Figure 9**), suggesting that Neat1_2 is partially involved in inflammatory regulation of primary sensory neurons in neuropathic pain. Furthermore, Neat1_1 overexpression in intact rats led to the upregulation of these genes (**Figure 4C**). Thus, Neat1 increased the expressions of inflammatory genes in the DRG during neuropathic pain. On the other hand, Neat1 overexpression did not induce the expression of neuronal injury marker, ATF3, while caused the downregulation of several ion channels as seen after nerve injury (Na_V_1.1, Na_V_1.6 and K_V_1.2) (8, 38, 39), indicating that Neat1 overexpression partially recapitulated the gene expression changes after nerve injury (**Supplementary Figure 10**).

In contrast, genes downregulated by Neat1 in neuropathic pain included those associated with neuronal function, such as voltage-gated sodium channels (Na_V_1.1, Na_V_1.6, and Na_V_1.9) and calcium/calmodulin-dependent kinase (CaMKIIα) (**Supplementary Table 5**). Consistent with previous reports (38–40), these genes were decreased in the L5 DRG following nerve injury, but these downregulations were counteracted by Neat1 knockdown (**Supplementary Figure 11**). However, these gene expressions did not appear to be related to the pro-nociceptive effect of Neat1 because the downregulation of these channels reportedly suppressed neuropathic pain (41–43).

### Neat1 coordinates the expression of inflammatory genes in the DRG *via* RNA-RNA interaction-dependent and -independent mechanisms

**Figure 5:**
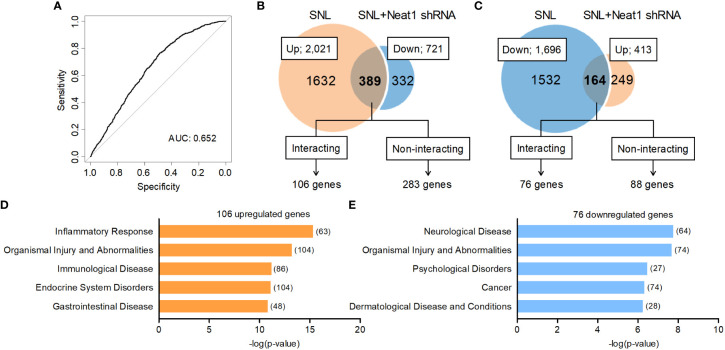
Neat1 coordinates the expression of inflammatory genes in the DRG *via* RNA-RNA interaction-dependent and -independent mechanisms. **(A)** An ROC curve for Neat1-regulated genes in neuropathic pain (553 genes). *P* < 0.001 compared with the randomized control data set. **(B, C)** Outline of interacting or non-interacting genes with Neat1 predicted by RIblast in genes upregulated [**(B)**; 389 genes], and genes downregulated [**(C)**; 164 genes] through Neat1. An interaction energy threshold -8000 kcal/mol was used to extract target genes for Neat1 (17). The most relevant diseases and disorders to each gene set was assessed using IPA. **(D, E)** Top five of diseases and disorders associated with 106 upregulated genes **(D)** and 76 downregulated genes **(E)** that interact with Neat1 were assessed by IPA. The number of genes involved in the pathways was shown in brackets.

Because Neat1 was mainly localized to the nucleus whereas mature microRNA generally localized to the cytoplasm, microRNAs might not be the main molecular target of Neat1 in the DRG. Alternatively, Neat1 was predicted to interact with mRNAs in the nucleus (44). Therefore, we examined the potential significance of the Neat1-mRNA interactome in changes in gene expressions after nerve injury. The interaction energy of Neat1 to each mRNA was calculated *in silico* using RIblast, a computational lncRNA-RNA interaction prediction tool based on nucleotide sequences and secondary structures (17). The dependence of Neat1-mediated changes in gene expressions after nerve injury (553 genes) on the interaction potential between Neat1 and each mRNA was assessed using an area under the receiver operating characteristic curve (AUROC). The AUROC score of the interaction energy against expression changes was 0.652 (**Figure 5A**), suggesting that changes in gene expressions by Neat1 were at least partly mediated by the RNA interactome between Neat1 and Neat1-regulated mRNAs.

We found 106 out of 389 mRNAs upregulated and 76 out of 164 mRNAs downregulated through Neat1 in neuropathic pain were predicted to bind directly to Neat1 (**Figures 5B, C** and **Supplementary Table 6**). In gene ontology analysis using IPA, the diseases and disorders most relevant to the predicted Neat1-interacting genes that were upregulated (106 mRNAs) and downregulated (76 mRNAs) by Neat1 in neuropathic pain were inflammatory response and neurological disease (**Figures 5D, E**), respectively, similar to the analysis of genes whose expressions were changed by Neat1 knockdown regardless of Neat1 interactions (553 genes; **Figure 3E**). The predicted Neat1-interacting genes associated with inflammatory response (**Supplementary Table 6**) included many genes reported to be involved in spinal microglial activation (29, 33, 35, 36), including XCL1, LIF, COX-1, and NLRP3 that were upregulated through Neat1 (**Figure 4**).

### Neat1 increases expressions of inflammatory genes by stabilizing mRNAs

**Figure 6:**
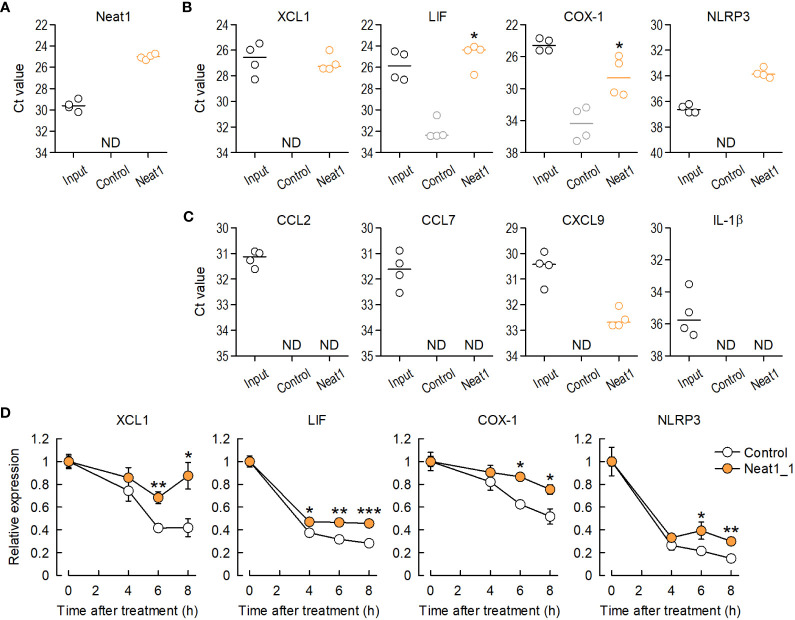
Neat1 interacts with inflammatory genes in the DRG *in vivo* through RNA-RNA interactions. **(A)** Enrichment of endogenous Neat1 assessed by RNA pull-down using DRG tissues with a negative-control or Neat1-specific probe. **(B, C)** *In vivo* interactions between Neat1 and mRNAs predicted to interact **(B)** or not to interact with Neat1 **(C)** were assessed by RNA pull-down. *P < 0.05 compared with the negative control probe, Mann-Whitney *U*-test (*n* = 4). ND = not detected. **(D)** The time course of mRNA expression changes of Neat1-interacting genes was examined in primary culture of DRG cells after actinomycin D treatment. *P < 0.05, **P < 0.01, and ***P < 0.001 compared with the control AAV, unpaired *t*-test (*n* = 5–7).

To investigate *in vivo* interactions between Neat1 and target mRNAs, an RNA pull-down assay was performed using DRG tissues where RNA-RNA interactions were preserved by fixation. RNA pull-down followed by RT-qPCR (18, 19) revealed that Neat1 pull-down highly enriched endogenous Neat1 (**Figure 6A**) and predicted interacting mRNAs (**Figure 6B**), indicating the *in vivo* interaction of Neat1 with target inflammatory gene mRNAs. In marked contrast, genes that were predicted to have the lowest potential to interact with Neat1 (Bcl2a1, Pthlh and Plac8) were not enriched (**Supplementary Figure 12**). Therefore, Neat1 might increase target mRNA expressions at least in part through RNA-RNA interactions. Interestingly, genes that were predicted not to interact with Neat1 were most associated with immunological disease (**Supplementary Figure 13**; **Supplementary Table 7**). These immune-related genes included several molecules related to microglial activation, including, CCL2, CCL7, CXCL9, and IL-1β. As expected, these mRNAs were not enriched by Neat1 pull-down (**Figure 6C**). These results suggested that Neat1 orchestrated the expressions of key inflammatory genes by RNA-RNA interaction-dependent and -independent mechanisms in primary sensory neurons.

To further investigate whether Neat1 increases expressions of inflammatory genes by regulating decay of target mRNAs, RNA-decay assay was performed to examine decay levels of target mRNAs by blocking transcription of mRNA using actinomycin D and DRB. Neat1 overexpression in primary culture of DRG cells suppressed decay of Neat1-interacting mRNAs after actinomycin D or DRB treatment (**Figure 6D** and **Supplementary Figure 14**). Therefore, Neat1 can increase expression levels of interacting inflammatory genes by stabilizing those mRNAs.

### Neat1 inhibition in primary sensory neurons represses neuroinflammation in the spinal cord

**Figure 7:**
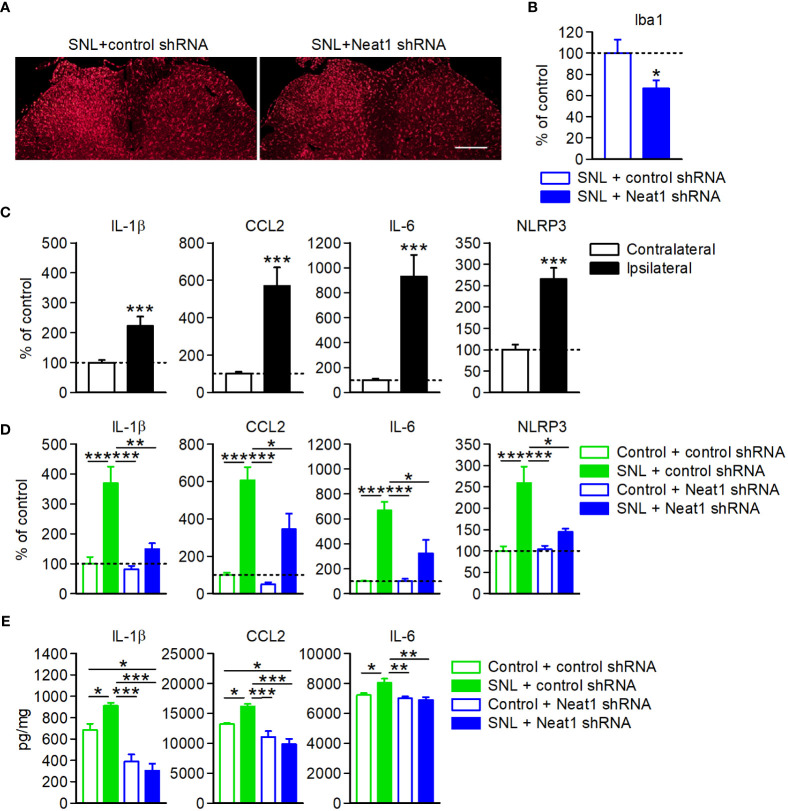
Neat1 in primary sensory neurons contributes to neuroinflammation in the spinal cord. **(A, B)** Representative images **(A)** and the intensity **(B)** of Iba1 immunofluorescence in the spinal cord 14 days after SNL in rats injected with the AAV vector encoding control or Neat1 shRNA. Scale bar, 200 µm. *P < 0.05 compared with the control shRNA, unpaired *t*-test (*n* = 5–6). **(C–E)** mRNA **(C, D)** and protein **(E)** expression levels of proinflammatory genes in the L5 dorsal spinal cord 14 days after SNL **(C)** and 14 days after SNL in rats injected with control or Neat1 shRNA AAV **(D, E)**, as a percentage of the expression level in the L5 spinal cord of intact rats injected with control shRNA AAV vector. ***P < 0.001, compared with the contralateral side, Mann-Whitney *U*-test [**(C)**; *n* = 10]. *P < 0.05, **P < 0.01, and ***P < 0.001, two-way ANOVA followed by Tukey’s test [**(D)**; *n* = 8–9 and **(E)**; *n* = 6].

It has been reported that inflammatory mediators regulated by Neat1 (**Figure 4**) are induced in primary sensory neurons upon nerve injury and are involved in spinal microglial activation, leading to neuropathic pain (23–29, 31, 33–35, 45). Neat1 knockdown in primary sensory neurons suppressed the peripheral nerve injury-induced activation of microglia, immune cells resident in the central nervous system, as indicated by the reduction of the intensity of Iba1 immunofluorescence (**Figures 7A, B**). Consistent with this, increased expressions of proinflammatory factors (IL-1β, CCL2, IL-6, and NLRP3) in the dorsal spinal cord of SNL rats (**Figure 7C**) were also suppressed by Neat1 downregulation in primary sensory neurons (**Figure 7D**). Upregulation of cytokines (IL-1β, CCL2, and IL-6) in the dorsal spinal cord was also suppressed by Neat1 knockdown at protein levels (**Figure 7E**).

**Figure 8:**
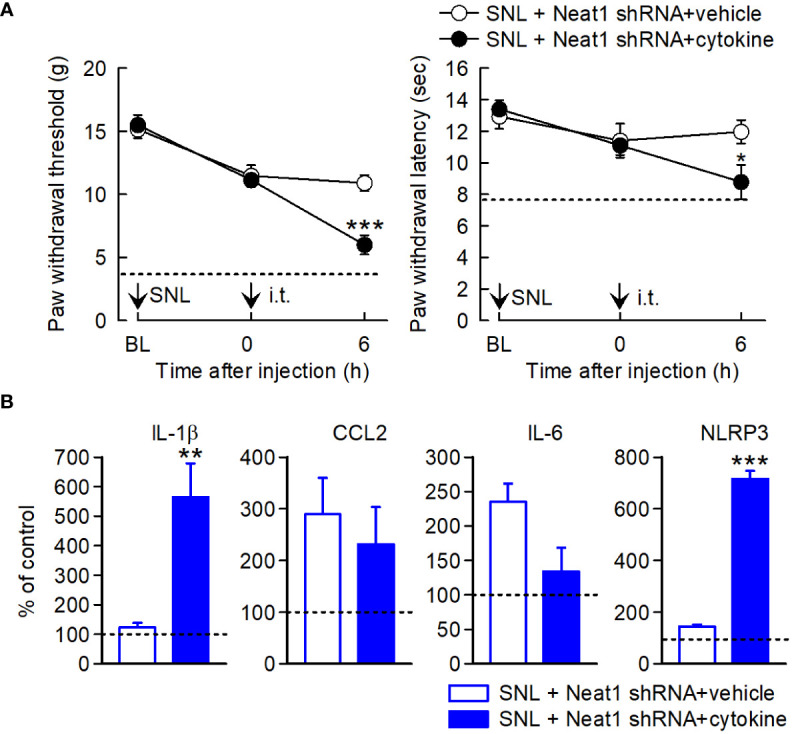
Neuropathic pain relapses by inflammatory mediators regulated by Neat1. **(A)** Paw withdraw thresholds and latencies to mechanical and thermal stimuli, respectively, were evaluated. Neat1 shRNA AAV was injected 7 days before SNL. Cytokines (CCL2, CCL7, CXCL9, IL-1β, and LIF) were intrathecally injected 14 days after SNL. *P < 0.05 and ***P < 0.001 compared with the vehicle injection (*n* = 7), Mann-Whitney *U*-test for mechanical hypersensitivity, unpaired *t*-test for thermal hypersensitivity. The dot lines represent the mean values of paw withdraw thresholds and latencies at day 14 after SNL in rats injected with control shRNA AAV. **(B)** mRNA expression levels of proinflammatory genes in the L5 dorsal spinal cord 6 h after cytokine treatment, as a percentage of the expression level in the L5 spinal cord of intact rats injected with control shRNA AAV vector, **P < 0.01 and ***P < 0.001 compared with the vehicle injection, Mann-Whitney *U*-test (*n* = 7). i.t. indicates intrathecal administration.

**Figure 9:**
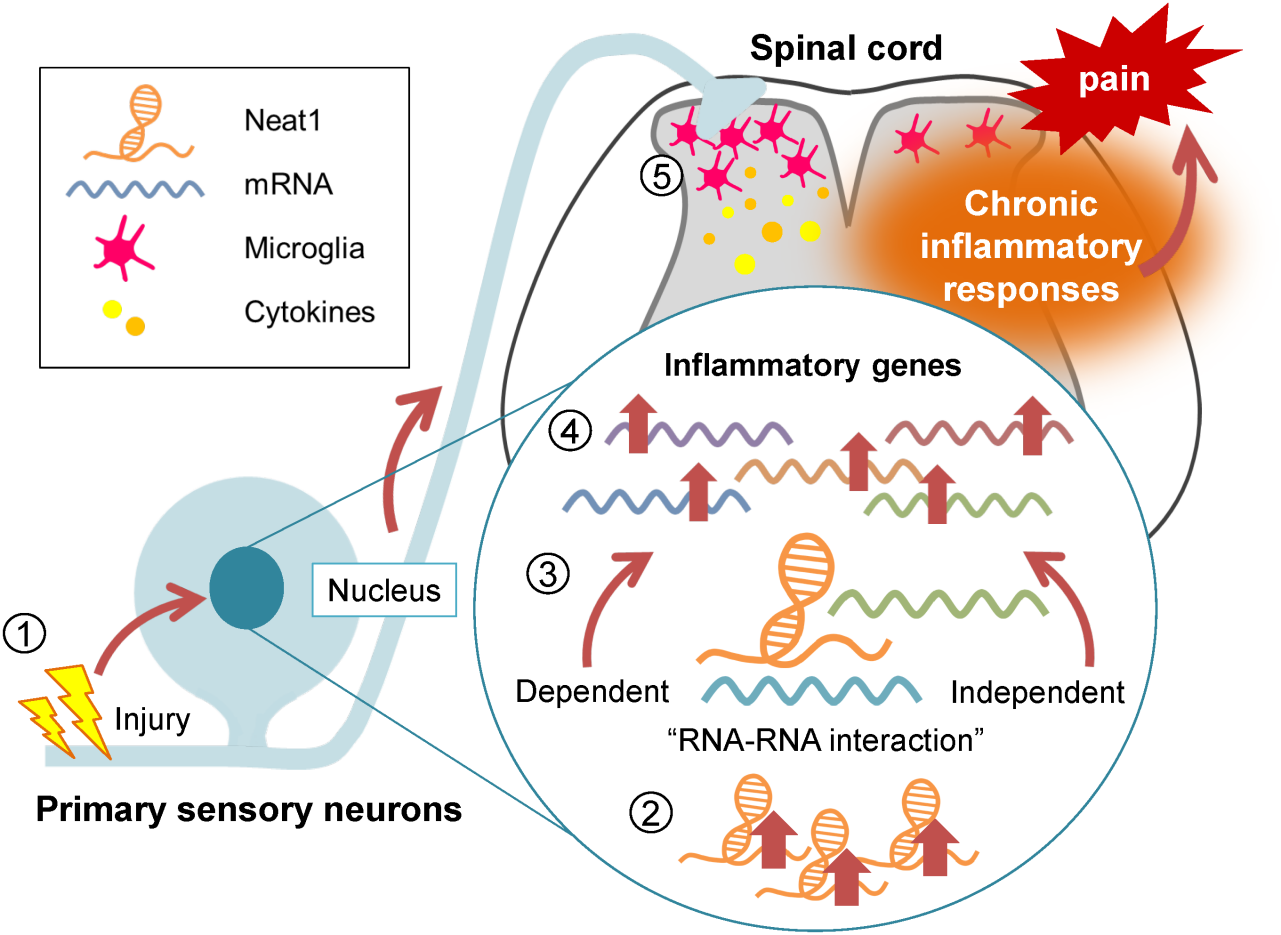
Neat1 regulates the proinflammatory functions of the DRG cells in neuropathic pain. Diagram summarizing the regulatory role of Neat1 in spinal inflammation in peripheral neuropathic pain. Nerve injury-induced upregulation of Neat1 in the DRG increases the expressions of inflammatory genes through RNA-RNA interaction-dependent and -independent mechanisms. These upregulations of inflammatory genes might result in microglial activation and upregulation of pro-inflammatory cytokines in the spinal cord, leading to chronic neuropathic pain.

Finally, we investigated whether neuropathic pain relapsed by Neat1-regulated inflammatory mediators in SNL rats with Neat1 downregulation. Cytokine cocktail including CCL2, CCL7, CXCL9, IL-1β, and LIF was intrathecally injected 14 days after SNL in rats injected with Neat1 shRNA AAV. Treatment of cytokine cocktail caused the recurrence of mechanical and thermal hypersensitivity (**Figure 8A**) and increased IL-1β and NLRP3 expressions, but not CCL2 and IL-6 (**Figure 8B**). Therefore, proinflammatory changes induced by Neat1 in primary sensory neurons led to immune cell-mediated spinal inflammatory responses (**Figure 9**).

## Discussion

Here, we showed that Neat1 lncRNA organized the proinflammatory gene expressions in the DRG during neuropathic pain through RNA-RNA interaction-dependent and -independent mechanisms (**Figure 9**). Neat1 coordinated the expressions of diverse inflammatory genes known to cause microglial activation, which are critical for the pathology of peripheral neuropathic pain. Indeed, Neat1 mediated the increased productions of various cytokines (CCL2, CCL7, CXCL9, XCL1, IL-1β, and LIF), which are reportedly involved in spinal microglial activation, in the DRG after nerve injury. CCL2, CCL7 and CXCL9, and IL-1β were shown to activate spinal microglia and their blockade suppressed both microglial activation and neuropathic pain (24, 27, 46–48). However, activation of spinal microglia by CCL2 may not be mediated through a direct effect of CCL2 on microglia because a large body of literature have shown no CCL2 receptor (CCR2) expression in spinal microglia (49), although some groups reported a CCR2 expression in spinal microglia (50, 51). Intrathecal administration of XCL1 induced microglial marker expression and mechanical hypersensitivity, and the blockade of XCL1 reduced neuropathic pain (29). LIF was upregulated in the DRG after nerve injury (45) and its overexpression induced the spinal microglial proliferation (33). However, cell types expressing XCL1 and LIF in the DRG remain unclear. In addition to these secretory signaling molecules, Neat1 also regulated intracellular regulatory molecules of inflammation, such as COX-1 and NLRP3. It is reported that COX-1 co-localized with prostaglandin E synthetase in primary sensory neurons, and prostaglandin E_2_ contributed to phosphorylation of p38 MAP kinase and microglial activation after nerve injury (34, 35). Neat1 also increased the expression of NLRP3, a major component of the inflammasome, whose activation induces the release of IL-1β (36). NLRP3 was increased in primary sensory neurons and macrophages in chemotherapy-induced peripheral neuropathy, inducing mechanical hypersensitivity (37). Interestingly, Neat1 was also reported to be associated with and promote activation of the NLRP3 inflammasome (52). Thus, Neat1 organizes the proinflammatory function of DRG cells upon nerve injury, leading to microglial activation. However, treatment of cytokine cocktail including CCL2, CCL7, CXCL9, IL-1β, and LIF 14 days after SNL in rats injected with Neat1 shRNA AAV caused the recurrence of neuropathic pain, while increased only IL-1β and NLRP3, possibly because a portion of proinflammatory cytokines regulated by Neat1 was administered in this study. Of note, Neat1 also upregulated inflammatory genes involved in neurogenic inflammation. For example, prostaglandins, COX-1-mediated products, and CCL2 in primary sensory neurons were reported to contribute to peripheral tissue inflammation (53, 54). Therefore, Neat1 dysregulation might promote the deterioration of inflammatory diseases involving primary sensory neurons, although roles of Neat1 in non-neuronal cells in the DRG cannot be excluded.

Interestingly, Neat1 expression was also shown to be dysregulated in several neurodegenerative diseases accompanied by neuroinflammation. In the early phase of amyotrophic lateral sclerosis, the increased expression of Neat1_2 in motor neurons was reported in patients (55). Elevated Neat1 levels were also detected in postmortem brain samples and the peripheral blood of patients with Parkinson’s disease (56). Neat1 activated the NLRP3 inflammasome and upregulated proinflammatory cytokines by sponging several microRNAs in Parkinson’s disease. Thus, Neat1 might be a key organizer of neuroinflammation in neuronal diseases, including neuropathic pain.

Both RNA-RNA interaction-dependent and -independent mechanisms of Neat1 were cooperatively engaged in the regulation of inflammatory gene expression in the DRG. In this study, a comprehensive analysis of the Neat1-RNA interactome using RIblast identified various pain-relevant mRNAs, which were verified by *in vivo* RNA pull-down assay. AUROC analysis of whole transcriptome changes and interaction energy with mRNAs determined by RIblast demonstrated that gene expression changes mediated by Neat1 were predictable by the interaction potential of each mRNA to Neat1, indicating the involvement of RNA interactions in gene expression changes mediated by Neat1. Consistent with this, Neat1 interacted with XCL1, LIF, COX-1, and NLRP3 mRNAs *in vivo* in the DRG and increased their expressions. Furthermore, RNA decay assay revealed that Neat1 stabilized these Neat1-interacting mRNAs. Indeed, RNA-RNA interactions between lncRNAs and target RNAs in the nucleus can induce both positive and negative regulation for mRNA homeostasis (stabilization and degradation, respectively) (57). Antisense lncRNAs reportedly stabilized and upregulated its sense counterpart mRNA. For instance, PDCD4-AS1 lncRNA promoted the stability of PDCD4 mRNA by forming an RNA duplex, which prevented its binding to HuR, an RNA decay factor inside the nucleus (58). RNA duplex formation with lncRNA also stabilized mRNA by preventing RNase-mediated degradation (59). Neat1 was also reported to upregulate TNFRSF1B (60) or ELF3 mRNA by promoting the stabilization of its mRNA (61). In this study, TNFRSF1B was identified as a Neat1-binding gene (**Supplementary Table 6**) that was upregulated through Neat1 (**Supplementary Table 4**), whereas ELF3 expression was unchanged, most likely because of its very low basal expression in the DRG. In contrast to the stabilization of interacting mRNAs, TINCR lncRNA induced the decay of epidermal differentiation mRNAs through RNA-RNA interactions (62). Antisense lncRNAs have been shown to decrease their host mRNA expressions. Therefore, Neat1 regulates specific proinflammatory genes by stabilizing mRNAs in the DRG through interactions with target mRNAs. In addition to interacting mRNAs, Neat1 also upregulated the expressions of non-interacting inflammatory genes in the DRG. Neat1 has been reported to regulate gene expression by acting as a scaffold lncRNA to sequester or recruit/guide specific proteins, such as transcription factors and splicing factors (63–66). Therefore, Neat1 may also increase the expression of inflammatory genes through binding to gene regulatory proteins in the DRG.

## Conclusions

In summary, Neat1 provides extensive control over the proinflammatory functions of the DRG cells including primary sensory neurons after nerve injury by RNA-RNA interaction-dependent and -independent mechanisms. However, this study has a limitation for potential translation as most patients with chronic pain are elderlies, while young rats were used in this experiment. Further studies of Neat1 functions will provide further insights into primary sensory neuron-mediated inflammatory responses that underlie chronic intractable neuropathic pain as well as other chronic inflammatory disorders.

## Data availability statement

The data presented in the study are deposited in the GEO repository, accession number GSE228730.

## Ethics statement

All animal experimental procedures were reviewed by the Nippon Medical School Animal Care and Use Committee, approved by the President of the Nippon Medical School (Approval number, 27-037 and 2020-042), and performed in accordance with the guidelines of the International Association for the Study of Pain (67).

## Author contributions

MM, AS, and HS designed the study and wrote the manuscript. MM and AS conducted experiments and prepare figures. YM and TO generated AAV vectors. TF and MH performed computational analysis. All authors contributed to the article and approved the submitted version.
