# Supplementary material for: Neat1 lncRNA organizes the inflammatory gene expressions in the dorsal root ganglion in neuropathic pain caused by nerve injury
Source: Front Immunol. 2023 Aug 8;14:1185322. doi: 10.3389/fimmu.2023.1185322 (PMC10442554; doi:10.3389/fimmu.2023.1185322)
Supplement: Supplementary file 1 [file DataSheet_1.pdf]

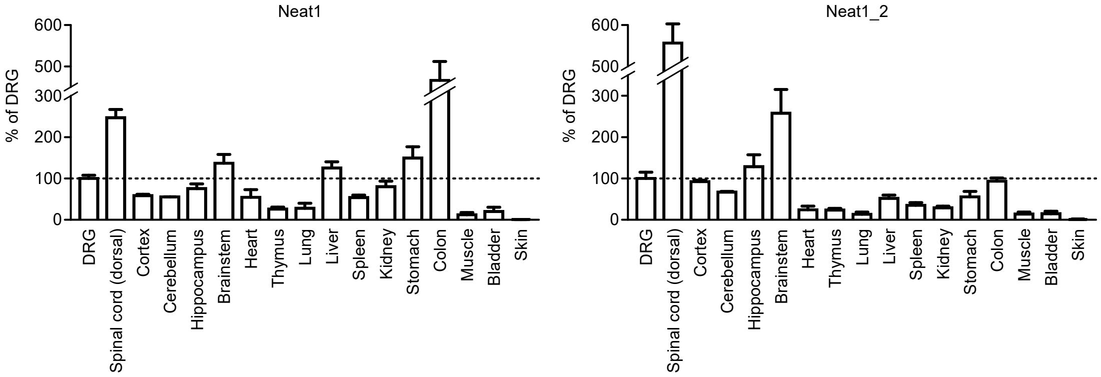

Supplementary Figure 1. Neat1 is highly expressed in the nervous system in rats.

Expression levels of Neat1 and Neat1_2 were examined in various intact rat tissues using qPCR (*n* = 3).

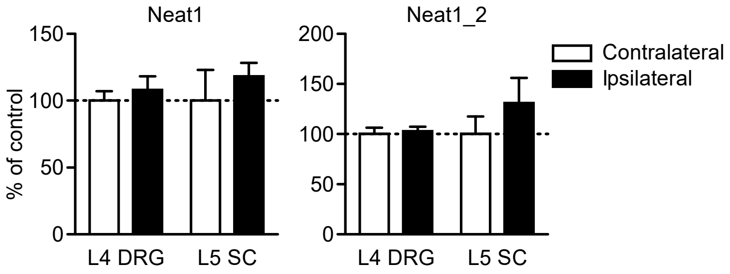

Supplementary Figure 2. Neat1 is not changed in the injury-spared L4 DRG and L5 dorsal spinal cord after nerve injury. Expression levels of Neat1 and Neat1_2 were examined in the injury-spared L4 DRG and L5 dorsal spinal cord (SC) 14 days after SNL using qPCR (n = 4–5).

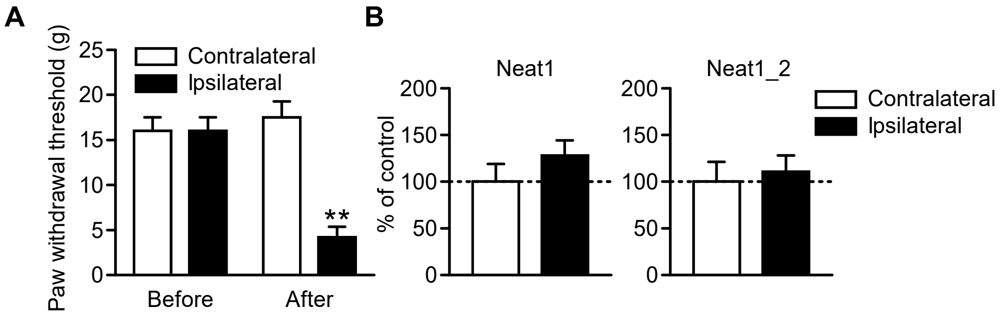

Supplementary Figure 3. Neat1 expression is unchanged in the L5 DRG during inflammatory pain. (**A**) Paw withdraw thresholds to mechanical stimuli were evaluated before and 7 days after injection of complete Freund's adjuvant (CFA). $^{**}P < 0.01$ compared with contralateral side ($n = 4$), paired *t*-test. (**B**) Expression levels of Neat1 and Neat1_2 were examined in the L5 DRG 7 days after injection of CFA ($n = 4$).

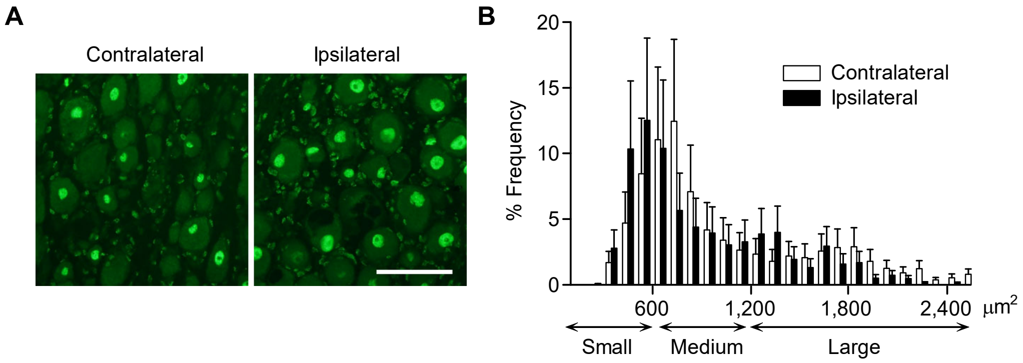

Supplementary Figure 4. Neat1_2 is mainly localized in the nucleus of DRG neurons.

(**A**) Representative images of *in situ* hybridization for Neat1_2 expression in the L5 DRG on the contralateral and ipsilateral sides of rats 14 days after SNL (n = 4). (**B**) Size distribution of Neat1_2-positive neurons in the L5 DRG on the contralateral and ipsilateral sides of rats examined by *in situ* hybridization (n = 4).

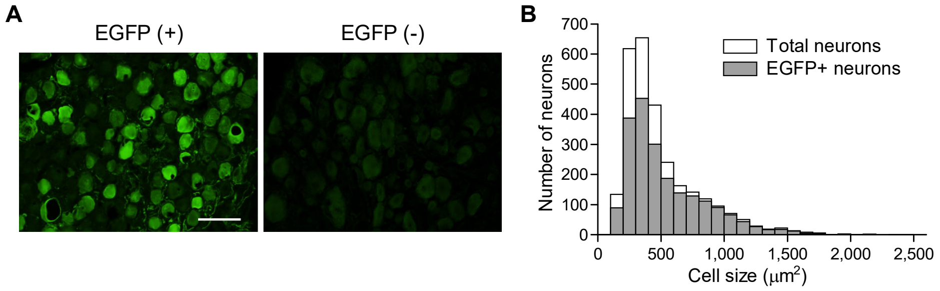

Supplementary Figure 5. Injection of the AAV vector induced EGFP expression in the L5 DRG neurons. (**A**) Representative images of EGFP immunofluorescence in the L5 DRG with (EGFP+) and without (EGFP-) AAV vector injection. Scale bar, 100 μm. (**B**) Size distribution of GFP-positive neurons 7 days after AAV vector administration. Eight DRG sections obtained from individual rats were counted (total 2 rats).

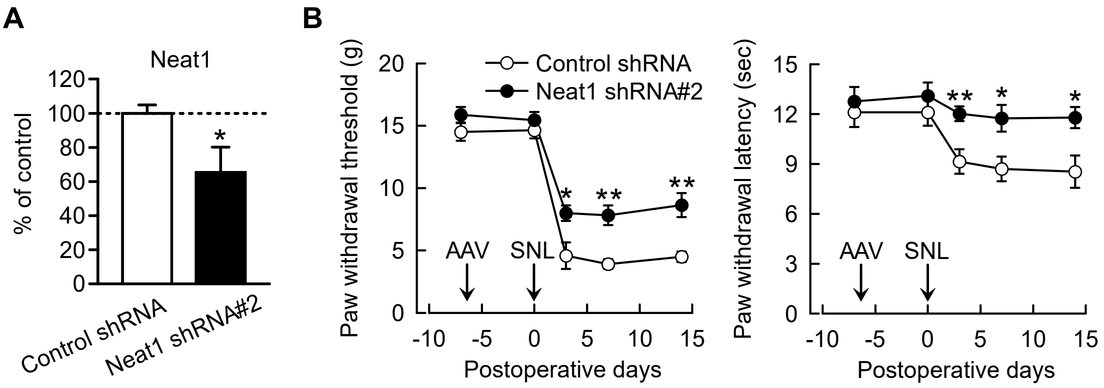

Supplementary Figure 6. Neat1 downregulation in primary sensory neurons using another shRNA also suppresses neuropathic pain. (**A**) Expressions of Neat1 in the L5 DRG 14 days after SNL in rats injected with control or Neat1 shRNA#2 AAV. $*P < 0.01$ compared with the control shRNA AAV, Mann-Whitney *U*-test (n = 5–6). (**B**) Paw withdraw thresholds and latencies to mechanical and thermal stimuli, respectively, were evaluated. Control or Neat1 shRNA#2 AAV was injected 7 days before SNL. $*P < 0.05$ and $**P < 0.01$ compared with SNL rats with control shRNA (n = 7–8), unpaired *t*-test.

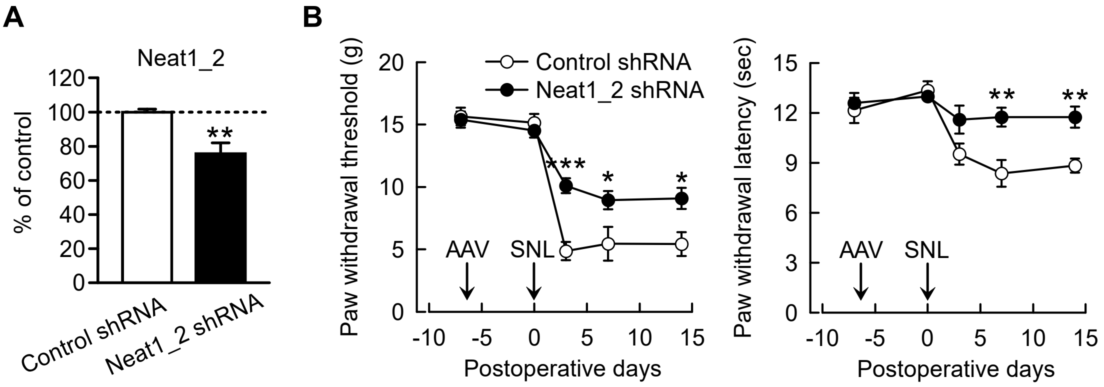

Supplementary Figure 7. Neat1_2 in primary sensory neurons contributes to neuropathic pain. (**A**) Expressions of Neat1_2 in the L5 DRG 14 days after SNL in rats injected with control or Neat1_2 shRNA AAV. $^{**}P < 0.01$ compared with the control shRNA AAV, Mann-Whitney *U*-test ($n = 5–6$). (**B**) Paw withdraw thresholds and latencies to mechanical and thermal stimuli, respectively, were evaluated. Control or Neat1_2 shRNA AAV was injected 7 days before SNL. $^{*}P < 0.05$, $^{**}P < 0.01$, and $^{***}P < 0.001$ compared with control shRNA injection to SNL rats ($n = 7–8$), unpaired *t*-test.

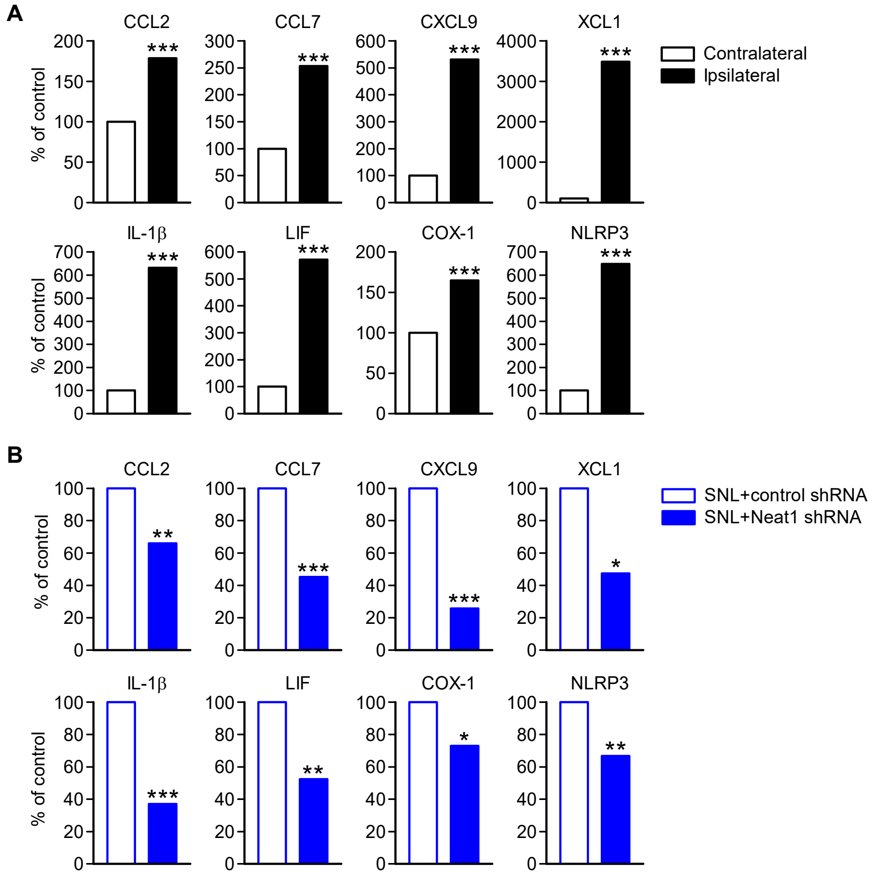

Supplementary Figure 8. RNA sequencing results of expression changes in multiple inflammatory genes after nerve injury. (**A and B**) Expressions of inflammatory genes were examined in the L5 DRG 14 days after SNL (**A**) and 14 days after SNL in rats injected with the AAV vector encoding control or Neat1 shRNA (**B**) using RNA sequencing. *$P < 0.05$, **$P < 0.01$, and ***$P < 0.001$, compared with contralateral side (**A**; $n = 3$), and the control shRNA injection to SNL rats (**B**; $n = 3$), square root of Jensen-Shannon divergence adjusted by false discovery rate.

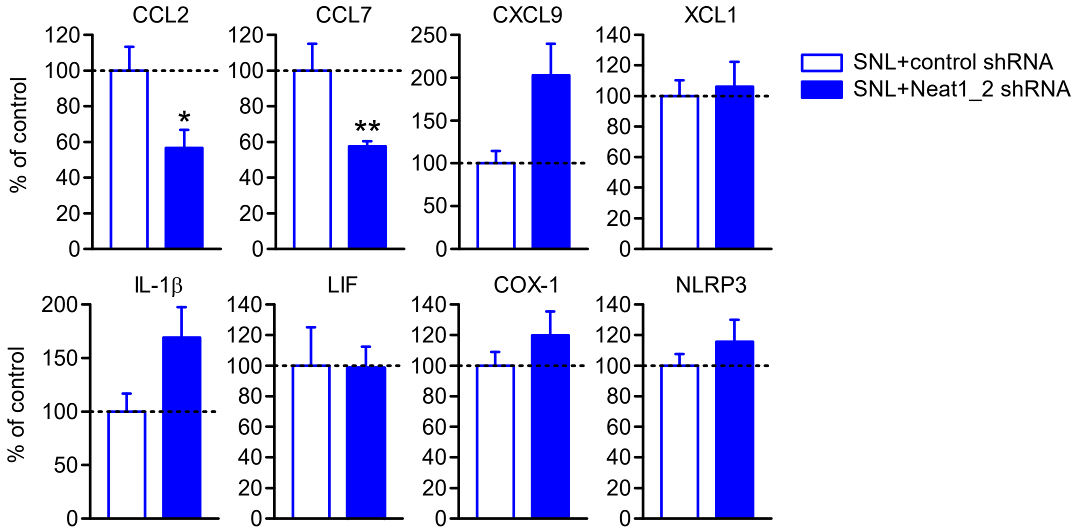

Supplementary Figure 9. Neat1_2 partially regulates the expressions of inflammatory genes in the DRG. Expressions of inflammatory genes in the L5 DRG 14 days after SNL in rats injected with the AAV vector encoding control or Neat1_2 shRNA were examined using qPCR. $*P < 0.05$ and $**P < 0.01$ compared with SNL rats with control shRNA injection, Mann-Whitney U-test ($n = 7$).

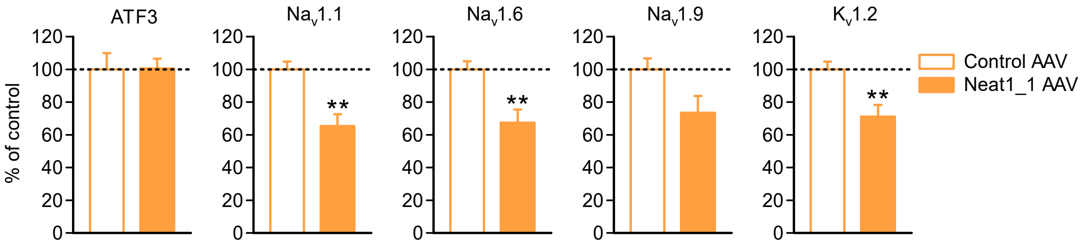

Supplementary Figure 10. Neat1 overexpression induces the downregulation of several ion channels in DRG neurons. Expressions of ATF3 and ion channels in the L5 DRG 14 days after control or Neat1_1 AAV injection were examined using qPCR. $^{**}P < 0.01$, compared with the control AAV injection to intact rats, Mann-Whitney-*U* test ($n = 8$).

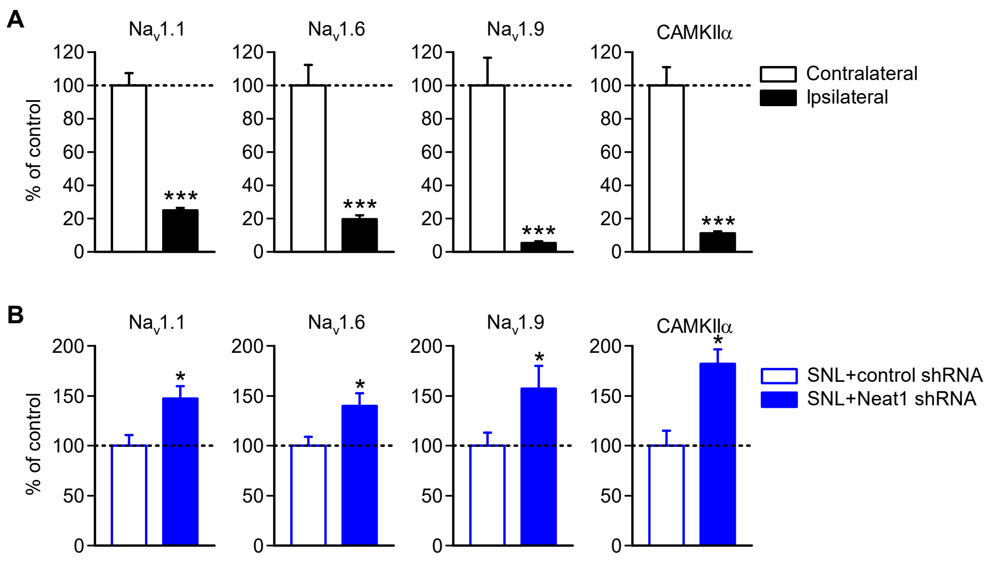

Supplementary Figure 11. Neat1 regulates genes associated with neuronal function in primary sensory neurons after nerve injury. (**A and B**) Expressions of genes associated with neuronal function in the L5 DRG 14 days after SNL (**A**) and 14 days after SNL in rats injected with the AAV vector encoding control or Neat1 shRNA (**B**). $*P < 0.05$ and $***P < 0.001$, compared with the contralateral side (**A**; $n = 6$) and with the control shRNA, Mann-Whitney-U test (**B**; $n = 5–8$).

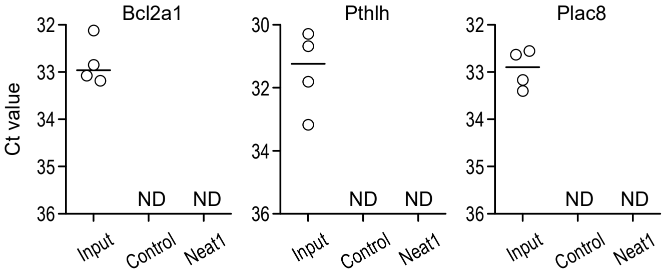

Supplementary Figure 12. Neat1 does not interact with genes predicted not to interact with Neat1 in RIblast. *In vivo* interactions between Neat1 and mRNAs predicted to have the lowest potential to interact with Neat1 were assessed by RNA pull-down ($n = 4$).

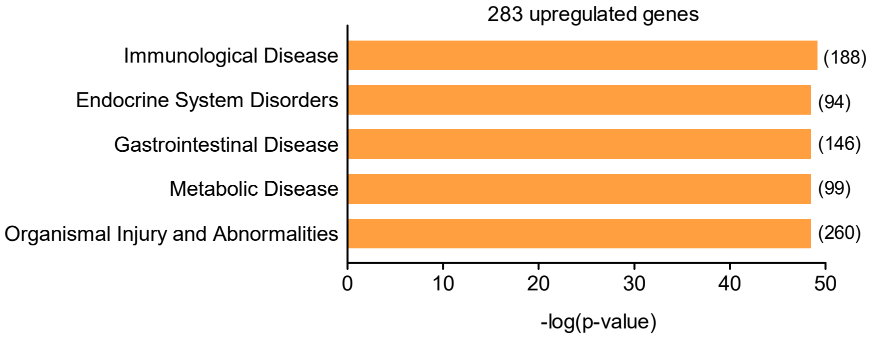

Supplementary Figure 13. Top five of diseases and disorders associated with upregulation of 283 genes in non-interacting genes with Neat1. The functions of associated with 283 genes in non-interacting genes with Neat1 were predicted by IPA. The number of genes involved in the pathways was shown in brackets.

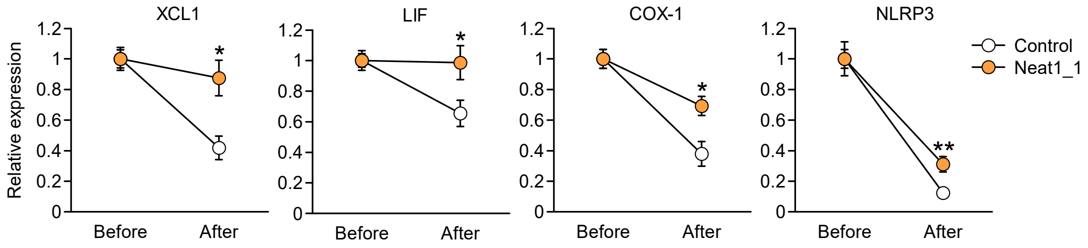

Supplementary Figure 14. Neat1 stabilizes Neat1-interacting mRNAs in primary sensory neurons. mRNA expression levels of Neat1-interacting genes in primary culture of DRG cells before and 8 h after DRB treatment. *$P < 0.05$ and **$P < 0.01$ compared with the control AAV, unpaired *t*-test (n = 5–7).

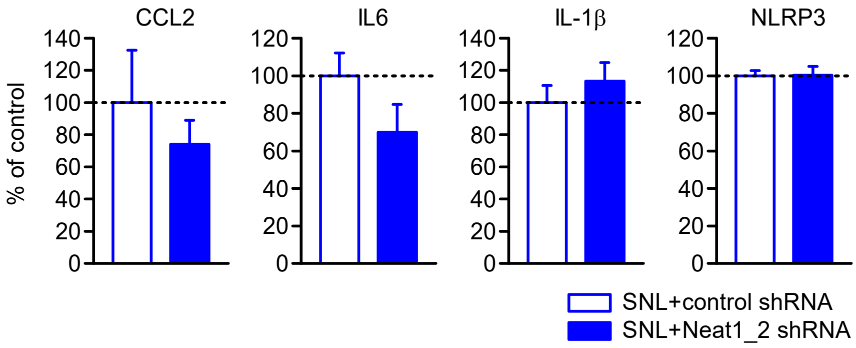

Supplementary Figure 15. Neat1_2 inhibition in primary sensory neurons does not repress neuroinflammation in the spinal cord. mRNA expression level of proinflammatory genes in the L5 spinal cord 14 days after SNL in rats injected with control or Neat1_2 shRNA AAV (*n* = 7).

Supplementary Table 1. List of primer sequences.

Primer sequences for identification of full length of rat Neat1 gene

Name	Forward primer (5'-3')	Reverse primer (5'-3')
Neat1 5´-RACE	CGACTGGAGCACGAGGACACTGA	AAATGGGTGCCGCAGGCCTGATG
Neat1 5´-RACE (nested PCR)	GGACACTGACATGGACTGAAGGAGTA	TCTCAACCTTCAGTCACCGC
Neat1_1 3'-RACE	CAGCTTGGCAATGGTGTATTGTCC	GCTGTCAACGATACGCTACGTAACG
Neat1_1 3'-RACE (nested PCR)	GTGCCAGTAAGGGATGTGCTTGGA	CGCTACGTAACGGCATGACAGTG
Neat1_2 3'-RACE	GCCCTCAGATTCCCCCTGAA	GCTGTCAACGATACGCTACGTAACG
Neat1_2 3'-RACE (nested PCR)	TAGACTCACAGAGGGGAGCC	CGCTACGTAACGGCATGACAGTG
Neat1_1 Fragment1	CGTGCTTCCTCTTCTGTGGT	TCTCAACCTTCAGTCACCGC
Neat1_1 Fragment2	GGAGAAGCGGGGCTAACTAT	GCACGGAACCTAGGCAAAGT
Neat1_1 Fragment3	CCAACACTGTGGGCTCTTGT	TCCAATGTGACCAGCAAGCA
Neat1_2 Fragment1	TCCCGTGACGAGTTTCCAAG	GGATGAGGGGCACACAGAAA
Neat1_2 Fragment2	CTTGCTGGTCACATTGGAATGGGG	GGATAGTTCAGCATGACGTTGGCC
Neat1_2 Fragment2 (nested PCR)	CTTGCTGGTCACATTGGAATGGGG	AAAGGCACGGAGGGAGCAAGCTAC
Neat1_2 Fragment3	AAAATGGTGTGACCAGGGCAT	GCCTGCATCTATCCACCCAC
Neat1_2 Fragment4	GCCTGCGGCACCCATTTCTTTGTA	ACGCAGATGCCGAAAGACAC
Neat1_2 Fragment5	CCTCTCACCCTGTAGATCCC	AGAAAGCTGCCTGAGAGTCAA
Neat1_2 Fragment6	GTGAATAGAACCCACCCCGA	AGGGTACTGCCTTGGTTTGG
Neat1_2 Fragment7	AACGCTCTGACTCTCCACTC	AGACGCACCAGAACCCTAGT
Neat1_2 Fragment8	CTGGTCGGCATAGTGAGTTCT	CTGACATCAAAGGGTGCTTGC
Neat1_2 Fragment9	GTCGGCAACTGTCAGCCATATTGC	TGGCCAAAGCACCTGGAATCT

Primer sequences for qPCR using SYBR Green

Gene	Forward primer (5'-3')	Reverse primer (5'-3')
Neat1	GGTGGAGTTGGTGCCAGTAA	TCTCAACCTTCAGTCACCGC
Neat1_2	TCCCGTGACGAGTTTCCAAG	CCTGGCTGACATCAAAGGGT
CCL2	TGATCCCAATGAGTCGGCTG	TGGACCCATTCCTTATTGGGG
CCL7	GTGTCCCTGGGAAGCTGTTAT	TCAGGGCTTTGGAGTTGAAGTT
CXCL9	TGGAGTTCGAGGAACCCTAGT	CAGTTAGGGCTTGGGGCAAA
XCL1	CCACAAGCCAAATGGGTGAA	CTCTGGGCTTGTGTGGGAAT
LIF	TGTGCCCCTACTGCTCATTC	GGTGGCATTTACAGGGGTGA
COX-1	CGGGCCCCAACTGTACTATC	CCAGATCCAGTACCCGTGTG

Assay ID for TaqMan qPCR

Gene	Assay ID
NLRP3	Rn04244620_m1
IL-1β	Rn00580432_m1
IL-6	Rn01410330_m1

Supplementary Table 2. List of lncRNAs registered in the rat gene database.
Genes marked in bold are differentially expressed after nerve injury
(fold change > 1.5 or < 0.67, P < 0.05, n = 3).

Gene	FPKM		Fold change	p_value
	CNT	SNL		
**Snhg11**	176.67	116.92	0.66	5.00E-05
**H19**	7.69	38.80	5.05	5.00E-05
Miat	114.02	81.61	0.72	5.00E-05
Gas5	54.06	67.40	1.25	4.03E-02
Snhg4	15.96	15.16	0.95	6.77E-01
Tug1	2.37	2.63	1.11	3.21E-01
Aptr	0.98	0.74	0.75	5.41E-01
Fendrr	0.39	0.40	1.03	1.00E+00

Supplementary Table 3. List of candidate homologues of human/mouse lncRNAs, which were differentially expressed after nerve injury.

Name	Location	FPKM		Fold change	p_value
		CNT	SNL		
Neat1	chr1:228133846-228137283	140.56	293.65	2.09	5.00E-05
Oip5-as1	chr3:117945532-117959069	34.47	21.43	0.62	5.00E-05
Nr2f2-as1	chr1:132528817-132552414	7.92	14.67	1.85	5.00E-05
Linc01135	chr5:117961200-117977705	7.00	16.20	2.32	5.00E-05
Linc00643	chr6:106646041-106657723	3.38	0.44	0.13	5.00E-05
Zeb2-as1	chr3:35196197-35196857	0.50	1.08	2.14	3.80E-02

Supplementary Table 4. List of 389 genes upregulated by SNL and downregulated by Neat1 knockdown (upregulated through Neat1).
Genes associated with the inflammatory response predicted by IPA were indicated.

Gene	FPKM		Fold change	p_value	FPKM		Fold change	p_value	Inflammatory responese
	CNT	SNL			SNL+Control	SNL+Neat1 shRNA			
Abcb4	0.55	3.18	5.73	5.00E-05	5.70	4.30	0.75	1.78E-02	○
Acap1	0.68	2.89	4.22	5.00E-05	11.99	3.95	0.33	5.00E-05	
Acp5	2.20	6.39	2.90	5.00E-05	12.45	8.67	0.70	8.65E-03	
Adamts1	7.93	23.60	2.98	5.00E-05	33.32	23.72	0.71	3.50E-04	○
Adamts12	2.22	4.66	2.10	5.00E-05	8.23	6.17	0.75	3.20E-03	○
Adap2	1.84	4.11	2.24	5.00E-05	7.62	5.87	0.77	4.28E-02	
Adcyap1	29.14	120.89	4.15	5.00E-05	90.30	60.81	0.67	1.45E-03	○
Adgre1	1.28	4.24	3.30	5.00E-05	9.74	6.43	0.66	5.00E-04	○
Adgrg5	0.25	2.10	8.30	5.00E-05	4.89	2.20	0.45	5.00E-05	
Aif1	13.83	53.16	3.84	5.00E-05	107.67	74.68	0.69	4.00E-04	○
Akna	2.12	4.56	2.16	5.00E-05	9.18	6.30	0.69	4.50E-04	○
Akr1b8	2.53	6.77	2.68	5.00E-05	14.97	10.22	0.68	1.35E-02	
Aldh1a3	3.59	7.45	2.07	5.00E-05	14.17	10.64	0.75	5.75E-03	
Ankrd1	0.63	11.06	17.69	5.00E-05	8.83	6.63	0.75	4.09E-02	
Anxa3	2.92	12.11	4.14	5.00E-05	21.75	15.95	0.73	1.40E-03	○
Apbb1ip	1.04	2.87	2.76	5.00E-05	5.66	3.76	0.66	4.15E-03	○
Apobr	0.68	2.73	4.02	5.00E-05	4.28	3.18	0.74	2.76E-02	
Apol3	3.24	5.44	1.68	5.00E-05	13.86	8.53	0.62	7.00E-04	
Arg1	1.56	31.89	20.41	5.00E-05	36.35	26.49	0.73	2.65E-03	○
Arhgap11a	0.76	2.22	2.94	5.00E-05	3.00	2.19	0.73	2.70E-02	
Arhgap15	1.27	3.63	2.84	5.00E-05	6.18	4.43	0.72	4.34E-02	
Arhgap30	0.68	3.26	4.81	5.00E-05	9.41	4.41	0.47	5.00E-05	
Arhgap4	2.26	5.23	2.32	5.00E-05	13.42	7.97	0.59	4.50E-04	
Arhgap9	1.57	8.65	5.51	5.00E-05	19.04	10.22	0.54	2.02E-02	○
Arhgdib	12.12	40.25	3.32	5.00E-05	88.27	56.68	0.64	5.00E-05	○
Arrdc4	10.23	18.44	1.80	5.00E-05	17.49	12.09	0.69	1.50E-04	
Arsi	0.77	1.66	2.16	5.00E-05	3.34	2.36	0.71	4.31E-02	
Artn	0.52	1.35	2.58	1.55E-03	3.33	2.12	0.64	1.75E-02	
Asb2	0.63	2.68	4.26	5.00E-05	5.90	2.99	0.51	5.00E-05	○
Asgr1	1.88	3.77	2.01	5.00E-05	10.08	7.11	0.70	2.77E-02	○
Asgr2	3.14	8.38	2.67	5.00E-05	23.54	14.79	0.63	4.50E-04	
Aurkb	0.40	3.01	7.55	5.00E-05	4.25	2.59	0.61	6.10E-03	
B3galt2	1.18	2.28	1.94	3.90E-03	2.19	1.30	0.60	2.41E-02	
Batf	0.17	1.07	6.21	3.25E-03	3.49	1.36	0.39	2.00E-04	○
Baz1a	1.03	2.75	2.68	5.00E-05	4.31	3.37	0.78	4.20E-02	
Bcl2a1	1.65	11.52	6.98	5.00E-05	29.61	14.14	0.48	5.00E-05	○
Bcl3	4.61	9.65	2.09	5.00E-05	14.90	11.74	0.79	3.99E-02	○
Bst2	20.16	52.89	2.62	5.00E-05	132.62	95.96	0.72	1.10E-03	○
Btg1	28.73	67.81	2.36	5.00E-05	101.96	81.54	0.80	1.52E-02	
Btk	0.24	1.24	5.14	5.00E-05	2.41	1.61	0.67	2.93E-02	○
C1s	20.72	74.19	3.58	5.00E-05	154.06	109.91	0.71	3.50E-04	○
C2	8.01	36.69	4.58	5.00E-05	61.48	42.89	0.70	5.00E-05	○
C3	0.79	7.32	9.29	5.00E-05	33.24	10.74	0.32	5.00E-05	○
C5ar1	1.74	3.34	1.91	1.00E-04	8.21	5.17	0.63	2.00E-03	○
Casp1	1.53	4.47	2.91	5.00E-05	8.74	5.32	0.61	2.25E-03	○
Casp8	1.37	2.92	2.13	5.00E-05	6.41	3.99	0.62	1.75E-03	○
Casq2	1.09	2.33	2.13	1.00E-04	3.85	2.19	0.57	1.75E-03	
Ccdc69	0.36	1.83	5.12	5.00E-05	4.02	1.94	0.48	2.50E-04	
Cck	0.72	12.46	17.42	5.00E-05	23.92	16.03	0.67	6.00E-03	○
Ccl17	0.58	20.80	35.57	1.01E-02	30.75	14.43	0.47	2.50E-04	○
Ccl2	16.50	29.51	1.79	5.00E-05	34.95	23.08	0.66	1.25E-03	○
Ccl22	0.08	3.36	42.20	5.00E-04	8.55	2.54	0.30	5.00E-05	○
Ccl7	2.68	6.80	2.53	5.00E-05	14.75	6.68	0.45	5.00E-05	○
Ccr5	0.62	5.13	8.25	5.00E-05	9.07	4.93	0.54	5.00E-05	○
Cd101	0.44	1.20	2.73	5.00E-05	1.65	1.04	0.63	8.35E-03	
Cd22	0.22	1.49	6.93	5.00E-05	2.81	1.67	0.59	1.85E-03	○
Cd244	0.36	1.88	5.23	5.00E-05	3.00	1.98	0.66	4.17E-02	○
Cd300lf	0.56	4.26	7.61	1.95E-03	7.23	4.82	0.67	1.56E-02	○
Cd300lf	41.29	3.46	0.08	5.00E-05	3.04	3.26	1.07	7.59E-01	○
Cd37	2.04	10.25	5.02	5.00E-05	26.19	14.36	0.55	5.00E-05	○
Cd38	1.11	2.75	2.47	5.00E-05	6.71	3.18	0.47	5.00E-05	○
Cd3d	0.10	1.33	13.53	2.65E-03	9.07	1.84	0.20	5.00E-05	○
Cd3e	0.12	1.69	14.43	1.50E-04	13.41	3.40	0.25	5.00E-05	○
Cd3g	0.37	2.23	5.99	3.00E-04	11.89	2.85	0.24	5.00E-05	○
Cd4	6.48	27.49	4.24	5.00E-05	55.19	37.83	0.69	5.00E-05	○
Cd40	0.27	1.04	3.79	7.50E-04	4.21	1.86	0.44	3.50E-04	○
Cd53	5.83	11.21	1.92	5.00E-05	22.14	11.77	0.53	5.00E-05	○
Cd6	0.39	2.21	5.70	5.00E-05	5.12	3.24	0.63	2.55E-03	○
Cd74	102.98	1143.84	11.11	5.00E-05	2185.09	1095.79	0.50	5.00E-05	○
Cd80	0.16	1.02	6.54	2.00E-04	3.01	1.50	0.50	2.90E-03	○
Cd93	3.09	5.49	1.78	5.00E-05	6.15	4.72	0.77	1.37E-02	○
Cdca7	0.70	1.23	1.77	1.90E-03	1.97	1.34	0.68	4.11E-02	
Cdh17	0.21	2.00	9.66	5.00E-05	2.96	2.03	0.69	1.81E-02	
Cdkn1a	3.34	42.24	12.63	5.00E-05	46.62	38.11	0.82	2.77E-02	○
Cep55	0.33	1.27	3.84	5.00E-05	1.42	0.93	0.66	3.68E-02	
Ciita	0.52	4.89	9.32	5.00E-05	9.06	3.99	0.44	5.00E-05	○
Cish	13.20	29.02	2.20	5.00E-05	25.76	20.99	0.81	4.27E-02	○

Cldn1	11.58	23.25	2.01	5.00E-05	40.70	33.89	0.83	4.57E-02	○
Cldn11	7.31	18.58	2.54	5.00E-05	71.15	54.35	0.76	6.95E-03	
Cldn4	0.15	21.59	144.76	5.00E-05	20.10	14.91	0.74	6.70E-03	
Clec12a	1.38	7.23	5.22	5.00E-05	11.90	7.00	0.59	1.10E-03	○
Clec2d2	0.14	2.95	21.66	5.00E-05	4.42	2.27	0.51	4.00E-04	
Clec2g	11.61	22.05	1.90	5.00E-05	34.66	26.96	0.78	5.55E-03	
Clec4a	0.59	2.04	3.43	2.00E-04	3.81	2.25	0.59	2.50E-02	○
Clec4a1	1.89	7.80	4.14	5.00E-05	15.15	10.23	0.68	5.10E-03	
Clec4a3	2.43	11.12	4.57	5.00E-05	21.00	14.67	0.70	9.15E-03	
Clec9a	0.30	1.10	3.71	5.00E-05	3.79	1.59	0.42	3.77E-02	○
Cmtm2a	0.34	10.17	30.07	5.00E-05	11.50	6.99	0.61	4.55E-02	
Col26a1	0.81	1.34	1.65	4.15E-03	0.98	0.60	0.61	3.02E-02	
Cotl1	46.16	86.06	1.86	5.00E-05	113.18	89.30	0.79	9.55E-03	○
Cpne8	8.30	16.42	1.98	5.00E-05	17.24	12.81	0.74	3.70E-03	
Crabp2	21.36	105.18	4.92	5.00E-05	131.26	98.12	0.75	2.00E-03	
Crisp1	0.38	63.86	169.00	5.00E-05	46.29	38.01	0.82	3.95E-02	
Csf2ra	4.46	13.08	2.93	5.00E-05	26.83	18.86	0.70	1.59E-02	○
Csf2rb	0.43	5.45	12.71	5.00E-05	9.71	5.64	0.58	5.00E-05	○
Csrnp1	1.46	8.07	5.53	5.00E-05	9.04	6.87	0.76	1.79E-02	
Csrp3	1.88	166.28	88.57	5.00E-05	124.07	86.59	0.70	5.00E-05	○
Cst7	0.41	3.93	9.54	5.00E-05	10.23	3.84	0.37	5.00E-05	
Ctla2a	4.45	10.88	2.44	5.00E-05	15.95	10.13	0.63	8.25E-03	
Ctse	0.53	3.02	5.73	5.00E-05	7.26	3.76	0.52	5.00E-05	○
Ctsw	0.19	1.49	7.91	1.50E-04	12.84	3.58	0.28	5.00E-05	○
Cxcl13	1.00	3.40	3.40	5.00E-05	51.86	2.81	0.05	5.00E-05	○
Cxcl9	6.22	33.04	5.31	5.00E-05	80.70	20.66	0.26	5.00E-05	○
Cxcr5	0.61	1.33	2.16	2.31E-02	0.79	0.34	0.44	2.90E-02	○
Cyba	11.79	35.99	3.05	5.00E-05	78.86	57.74	0.73	2.95E-03	○
Cybb	2.10	10.17	4.85	5.00E-05	17.97	13.70	0.76	4.45E-03	○
Cyp1b1	4.56	12.00	2.63	5.00E-05	14.97	12.42	0.83	4.68E-02	○
Cyp4b1	0.41	3.14	7.68	5.00E-05	3.75	2.26	0.60	4.25E-03	
Cysltr1	0.18	1.27	6.93	5.00E-05	1.34	0.94	0.70	3.94E-02	○
Cyth4	3.10	13.60	4.38	5.00E-05	21.93	17.24	0.79	1.51E-02	
Cytip	1.00	2.45	2.47	5.00E-05	6.57	3.26	0.50	1.00E-04	○
Dab2	5.81	15.81	2.72	5.00E-05	30.18	24.59	0.81	3.61E-02	○
Dapp1	0.37	2.60	6.99	5.00E-05	5.35	3.11	0.58	4.00E-04	○
Ddr2	4.28	8.28	1.93	5.00E-05	11.95	9.84	0.82	4.73E-02	○
Dennd1c	2.42	7.88	3.25	5.00E-05	18.43	9.63	0.52	5.00E-05	
Dhfr	11.48	74.45	6.48	5.00E-05	68.74	46.39	0.67	2.45E-02	○
Dock8	0.86	2.80	3.26	5.00E-05	5.76	3.59	0.62	5.00E-05	○
Dok2	1.08	2.58	2.38	5.00E-05	7.97	3.68	0.46	5.00E-05	○
Edn3	2.59	4.56	1.76	1.70E-03	6.88	5.13	0.75	3.10E-02	○
Efhd2	14.36	28.52	1.99	5.00E-05	43.59	35.97	0.83	3.90E-02	
Elf4	1.29	3.09	2.40	5.00E-05	4.97	3.61	0.73	5.55E-03	○
Emp1	17.28	74.09	4.29	5.00E-05	113.44	94.14	0.83	4.19E-02	
Enpp3	3.27	6.48	1.98	5.00E-05	13.75	10.84	0.79	3.04E-02	○
Epsti1	0.73	3.37	4.64	5.00E-05	11.37	4.74	0.42	5.00E-05	○
Ets1	4.57	7.97	1.74	5.00E-05	15.86	11.07	0.70	5.00E-05	○
Eya2	4.71	9.98	2.12	5.00E-05	20.00	15.97	0.80	3.25E-02	
Fam111a	0.41	1.14	2.77	5.00E-05	1.40	0.82	0.59	6.50E-03	
Fam180a	3.22	7.79	2.42	5.00E-05	19.26	14.00	0.73	6.40E-03	
Fap	0.89	3.74	4.20	5.00E-05	6.32	4.54	0.72	1.76E-02	
Fbn2	0.78	1.95	2.50	5.00E-05	2.70	2.13	0.79	3.80E-02	
Fcgr3a	0.86	6.35	7.40	5.00E-05	12.53	7.63	0.61	1.45E-03	
Fcnb	0.63	5.28	8.42	5.00E-05	12.53	2.61	0.21	5.00E-05	
Fcrla	0.22	1.91	8.72	5.00E-05	3.02	1.77	0.59	1.12E-02	○
Fem1c	2.79	5.62	2.02	5.00E-05	5.81	4.55	0.78	1.76E-02	
Fes	1.88	7.02	3.74	5.00E-05	9.36	6.83	0.73	6.55E-03	○
Fgd2	1.62	5.43	3.36	5.00E-05	9.91	6.95	0.70	2.90E-03	
Fgd3	0.76	1.90	2.49	5.00E-05	3.40	2.31	0.68	7.05E-03	
Fgr	0.49	3.76	7.75	5.00E-05	6.27	4.13	0.66	3.10E-03	○
Flnc	3.01	21.84	7.26	5.00E-05	18.43	14.50	0.79	7.20E-03	○
Fndc1	1.82	6.43	3.53	5.00E-05	15.57	11.07	0.71	1.50E-04	
Foxc1	2.13	3.98	1.87	5.00E-05	11.58	7.58	0.65	1.50E-04	
Foxp4	2.92	5.28	1.81	5.00E-05	11.68	9.04	0.77	1.32E-02	
G2e3	1.49	2.40	1.61	1.75E-03	2.21	1.67	0.76	4.98E-02	
Gal	18.80	1041.75	55.43	5.00E-05	892.27	679.73	0.76	7.20E-03	○
Galntl6	0.15	2.53	16.77	5.00E-05	2.62	1.52	0.58	1.48E-02	
Gbp2	9.22	38.23	4.15	5.00E-05	96.45	52.44	0.54	5.00E-05	○
Gbp5	2.91	8.43	2.90	5.00E-05	20.41	11.85	0.58	5.00E-05	○
Gimap7	0.86	2.14	2.50	1.00E-04	11.11	3.28	0.30	5.00E-05	
Gipc3	2.23	4.52	2.03	5.00E-05	6.18	4.63	0.75	3.02E-02	
Gjb2	3.02	5.64	1.87	5.00E-05	23.96	14.03	0.59	5.00E-05	○
Gmfg	6.31	19.16	3.04	5.00E-05	34.05	22.55	0.66	5.20E-03	○
Gna15	1.26	6.21	4.94	5.00E-05	12.58	8.89	0.71	8.75E-03	○
Gpr171	0.39	2.67	6.84	1.39E-02	6.16	2.03	0.33	3.50E-04	
Gpr31	0.16	1.21	7.74	5.00E-05	3.51	1.46	0.42	4.50E-04	○
Gpr4	1.64	2.68	1.63	1.50E-03	4.60	3.23	0.70	4.02E-02	○
Gpr65	0.31	1.11	3.62	2.15E-03	2.27	1.29	0.57	3.69E-02	○
Gpr84	0.07	1.05	14.19	2.10E-03	1.81	1.01	0.56	1.09E-02	○
Gsdmd	3.96	7.55	1.91	5.00E-05	14.38	11.54	0.80	4.76E-02	○
Gzmb	0.34	10.83	32.15	5.00E-05	11.43	4.62	0.40	5.00E-05	○
Hcls1	1.42	7.16	5.04	5.00E-05	16.56	8.77	0.53	5.00E-05	○

Hcn4	3.79	7.00	1.85	5.00E-05	10.52	7.90	0.75	3.90E-03	
Hcst	0.73	2.39	3.26	1.05E-02	12.87	5.71	0.44	7.00E-04	○
Hk2	1.43	3.62	2.52	5.00E-05	5.83	4.60	0.79	3.80E-02	
Hmgb2	5.55	17.19	3.10	5.00E-05	22.62	17.36	0.77	3.08E-02	
Hr	9.12	24.33	2.67	5.00E-05	26.35	21.83	0.83	3.39E-02	
Htra3	4.55	11.71	2.57	5.00E-05	26.25	21.34	0.81	4.63E-02	
Icam1	3.25	9.88	3.04	5.00E-05	24.19	17.74	0.73	1.60E-03	○
Ifi47	2.56	10.13	3.96	5.00E-05	30.37	12.59	0.41	5.00E-05	
Ifitm1	37.95	62.75	1.65	5.00E-05	158.99	115.76	0.73	8.00E-04	○
Igf2	6.18	10.24	1.66	5.00E-05	30.53	18.15	0.59	5.00E-05	○
Igfbp3	11.45	25.39	2.22	5.00E-05	35.50	29.13	0.82	3.70E-02	○
Igtp	7.19	16.59	2.31	5.00E-05	45.24	25.79	0.57	5.00E-05	○
Ikbke	2.90	5.43	1.87	5.00E-05	12.47	8.18	0.66	5.00E-05	○
Ikzf1	0.52	2.30	4.39	5.00E-05	5.15	2.58	0.50	5.00E-05	
Il10ra	0.91	3.52	3.86	5.00E-05	6.83	4.62	0.68	5.30E-03	○
Il13ra1	3.28	11.84	3.61	5.00E-05	11.18	8.19	0.73	3.50E-03	
Il17ra	8.51	14.10	1.66	5.00E-05	18.37	14.94	0.81	3.28E-02	○
Il1b	0.51	3.25	6.32	5.00E-05	8.73	3.23	0.37	5.00E-05	○
Il1rn	0.28	2.10	7.56	5.00E-05	3.02	1.94	0.64	1.70E-02	○
Il21r	0.43	2.68	6.22	5.00E-05	9.16	3.46	0.38	5.00E-05	○
Il2rb	0.11	2.30	20.63	5.50E-04	9.49	2.15	0.23	5.00E-05	○
Il2rg	2.50	7.39	2.96	5.00E-05	20.98	8.57	0.41	5.00E-05	○
Il4r	4.40	16.92	3.84	5.00E-05	22.78	18.18	0.80	1.87E-02	○
Irf1	5.14	11.00	2.14	5.00E-05	31.86	17.67	0.55	5.00E-05	○
Irf5	2.21	8.64	3.91	5.00E-05	12.50	9.59	0.77	2.19E-02	○
Irf8	1.89	11.16	5.89	5.00E-05	37.83	16.12	0.43	5.00E-05	○
Irgm	2.45	4.51	1.84	5.00E-05	13.48	8.95	0.66	5.00E-04	
Ism1	0.92	2.82	3.07	5.00E-05	12.44	8.33	0.67	1.30E-02	
Itgae	2.26	3.55	1.57	1.50E-04	6.62	5.02	0.76	2.99E-02	○
Itgal	0.30	3.08	10.11	5.00E-05	11.09	3.91	0.35	5.00E-05	○
Itgb2	0.96	6.97	7.24	5.00E-05	14.49	9.15	0.63	5.00E-05	○
Itgb7	0.48	2.14	4.49	5.00E-05	4.79	2.47	0.52	5.00E-05	○
Itgb8	17.79	44.98	2.53	5.00E-05	56.98	43.68	0.77	5.95E-03	○
Itln1	0.19	1.30	6.77	1.00E-04	2.40	1.51	0.63	3.29E-02	○
Jak2	8.34	21.08	2.53	5.00E-05	26.29	21.81	0.83	4.78E-02	○
Kcnj15	0.98	1.90	1.94	5.00E-05	3.13	1.86	0.60	6.50E-04	○
Kcnn4	5.60	10.25	1.83	5.00E-05	13.48	10.34	0.77	2.51E-02	○
Klf6	15.88	33.25	2.09	5.00E-05	36.26	30.26	0.83	4.59E-02	○
Klhl6	0.52	2.04	3.93	5.00E-05	5.38	3.66	0.68	1.36E-02	○
Klra1	0.42	1.85	4.38	5.00E-05	2.97	1.69	0.57	1.35E-03	
Klrk1	0.40	1.86	4.61	5.00E-05	9.51	3.60	0.38	5.00E-05	○
Lag3	0.46	1.01	2.19	5.30E-03	5.28	1.41	0.27	5.00E-05	○
Laptm5	8.38	28.57	3.41	5.00E-05	56.12	34.78	0.62	5.00E-05	○
Lat	0.92	1.69	1.84	8.20E-03	8.74	3.11	0.36	5.00E-05	○
Lat2	1.05	6.08	5.77	5.00E-05	9.19	6.36	0.69	3.88E-02	○
Lce1f	0.54	39.34	72.53	5.00E-05	19.92	12.80	0.64	5.40E-03	
Lck	0.99	1.83	1.86	5.00E-05	10.17	3.17	0.31	5.00E-05	○
Lcp1	45.63	136.58	2.99	5.00E-05	141.95	107.73	0.76	4.80E-03	○
Lcp2	1.68	4.44	2.64	5.00E-05	8.19	5.23	0.64	5.00E-04	○
Lfng	6.82	17.28	2.53	5.00E-05	26.24	21.10	0.80	3.07E-02	○
Lif	0.30	1.73	5.72	4.00E-04	3.39	1.77	0.52	3.15E-03	○
Lilrb4	1.36	9.32	6.87	5.00E-05	26.93	19.83	0.74	4.40E-03	○
Limd2	20.09	46.08	2.29	5.00E-05	71.05	50.02	0.70	1.53E-02	
LOC100910973	20.47	59.52	2.91	5.00E-05	234.45	104.80	0.45	5.00E-05	
LOC308990	0.37	1.50	4.07	5.00E-05	5.06	2.52	0.50	1.50E-04	
Lox	2.57	7.72	3.01	5.00E-05	14.11	10.62	0.75	7.90E-03	○
Lrrc18	0.72	4.38	6.05	5.00E-05	7.63	3.22	0.42	5.00E-05	
Lst1	0.44	2.54	5.73	3.15E-03	6.99	4.01	0.57	4.01E-02	
Ly6c	1.00	2.36	2.35	2.00E-03	7.85	3.28	0.42	2.00E-04	
Ly86	3.73	9.30	2.49	5.00E-05	18.26	10.51	0.58	2.50E-04	○
Lyc2	0.46	1.55	3.41	5.00E-05	4.54	1.65	0.36	5.00E-05	
Lyn	1.80	6.31	3.51	5.00E-05	10.25	7.83	0.76	1.49E-02	○
Lyz2	77.86	475.73	6.11	5.00E-05	1194.95	626.94	0.52	5.00E-05	
Maff	1.84	7.01	3.82	5.00E-05	10.81	8.36	0.77	4.20E-02	
Map3k8	0.95	2.87	3.02	5.00E-05	4.72	3.32	0.70	2.47E-02	○
Map4k1	2.01	9.25	4.60	5.00E-05	19.46	10.84	0.56	3.35E-03	○
Mapkapk2	13.79	24.03	1.74	5.00E-05	35.40	28.73	0.81	2.41E-02	○
Mdfic	4.40	7.64	1.74	5.00E-05	12.61	8.67	0.69	4.00E-04	
Mefv	1.17	2.82	2.41	5.00E-05	3.53	2.23	0.63	3.50E-03	○
Met	1.00	3.15	3.14	5.00E-05	4.85	3.57	0.74	3.15E-02	○
Mir205	0.08	2.46	28.99	5.00E-05	2.14	1.46	0.68	5.85E-03	
Mir221	0.41	2.01	4.87	4.00E-04	1.58	0.82	0.52	4.10E-02	
Mki67	0.69	3.40	4.89	5.00E-05	3.96	3.11	0.79	2.90E-02	○
Mmd	11.55	65.78	5.69	5.00E-05	60.56	47.69	0.79	7.70E-03	
Mnda	0.54	1.75	3.25	5.00E-05	6.19	2.86	0.46	5.00E-05	
Mob1a	5.77	10.17	1.76	5.00E-05	14.30	11.50	0.80	2.35E-02	
Mpeg1	3.63	21.65	5.97	5.00E-05	47.55	35.41	0.74	1.40E-03	○
Ms4a7	0.43	1.44	3.36	5.00E-05	4.48	2.31	0.51	9.00E-04	○
Msln	0.44	3.08	6.96	4.39E-02	8.62	4.48	0.52	5.00E-05	
Mycl	1.31	2.82	2.15	5.00E-05	6.01	3.72	0.62	6.80E-03	
Myo1f	1.10	4.83	4.38	5.00E-05	10.23	6.69	0.65	1.50E-04	○
Myrf	3.13	6.54	2.09	5.00E-05	7.29	5.46	0.75	5.45E-03	○
Napsa	0.84	8.39	10.03	5.00E-05	15.10	8.54	0.57	1.00E-04	

Ncf1	3.78	11.55	3.05	5.00E-05	35.61	16.14	0.45	5.00E-05	○
Ncf2	0.80	2.93	3.64	5.00E-05	5.36	2.50	0.47	2.50E-04	
Ncf4	1.20	5.32	4.43	5.00E-05	9.11	5.22	0.57	6.50E-04	○
Nck2	4.86	12.50	2.57	5.00E-05	13.69	10.80	0.79	2.95E-02	○
Nckap1l	2.29	6.07	2.65	5.00E-05	14.13	7.70	0.54	5.00E-05	○
Neurl3	0.46	2.79	6.09	5.00E-05	7.08	3.65	0.52	5.00E-05	
Nfil3	2.35	16.35	6.97	5.00E-05	16.02	11.05	0.69	1.50E-03	○
Nfkb2	4.47	9.80	2.19	5.00E-05	20.13	14.56	0.72	3.30E-03	○
Nfkbia	13.82	23.83	1.72	5.00E-05	49.35	34.13	0.69	1.00E-04	○
Nfkbiz	1.51	4.57	3.03	5.00E-05	7.81	5.48	0.70	2.17E-02	○
Nlrc4	0.21	1.11	5.34	5.00E-05	1.79	1.14	0.64	1.08E-02	○
Nlrp1a	0.48	1.24	2.60	5.00E-05	2.36	1.69	0.72	1.53E-02	○
Nlrp3	0.30	1.91	6.48	5.00E-05	3.89	2.60	0.67	3.95E-03	○
Npas2	0.47	1.28	2.71	5.00E-05	3.26	1.81	0.55	1.50E-04	
Ocln	0.79	1.71	2.16	5.00E-05	4.47	3.11	0.69	7.05E-03	○
Osr1	1.36	2.28	1.67	1.95E-03	9.23	6.57	0.71	1.46E-02	
P2ry13	0.37	1.28	3.48	4.30E-02	2.89	1.66	0.57	1.60E-03	○
Parp14	1.91	3.02	1.58	5.00E-05	6.52	5.09	0.78	1.95E-02	
Parp9	4.90	11.37	2.32	5.00E-05	20.83	16.64	0.80	4.71E-02	○
Parvg	0.86	3.62	4.21	5.00E-05	7.84	4.71	0.60	2.00E-04	
Pdcd2	15.93	37.07	2.33	5.00E-05	32.87	25.10	0.76	1.46E-02	
Pde7a	2.71	4.31	1.59	5.00E-05	5.04	3.50	0.69	6.65E-03	○
Pdgfra	4.65	9.11	1.96	5.00E-05	20.17	15.68	0.78	6.55E-03	○
Pi15	1.19	3.49	2.93	5.00E-05	7.99	5.29	0.66	1.18E-02	
Pik3ap1	0.56	1.55	2.78	5.00E-05	3.64	2.09	0.57	3.00E-04	○
Pik3r5	0.29	1.76	6.03	5.00E-05	3.40	2.19	0.64	2.55E-03	○
Pim1	3.22	10.75	3.33	5.00E-05	13.17	8.85	0.67	8.55E-03	○
Pla2g4a	0.47	1.72	3.67	5.00E-05	3.89	2.52	0.65	5.95E-03	○
Plac8	6.04	20.74	3.43	5.00E-05	90.17	46.77	0.52	5.00E-05	○
Plat	57.48	99.90	1.74	5.00E-05	131.83	109.29	0.83	4.24E-02	○
Pld4	4.48	24.60	5.49	5.00E-05	38.15	31.33	0.82	4.16E-02	○
Plek	1.72	8.98	5.23	5.00E-05	16.80	10.08	0.60	5.00E-05	○
Postn	2.69	14.04	5.23	5.00E-05	28.45	13.84	0.49	5.00E-05	○
Pou2f2	0.22	1.13	5.02	5.00E-05	2.49	1.68	0.68	2.18E-02	○
Prr5l	0.73	1.62	2.22	5.00E-05	2.99	1.82	0.61	6.50E-03	
Prss12	22.13	46.51	2.10	5.00E-05	50.72	21.45	0.42	5.00E-05	
Psd4	0.41	2.21	5.35	5.00E-05	4.61	2.66	0.58	5.00E-05	
Psmb9	5.75	15.65	2.72	5.00E-05	57.88	23.62	0.41	5.00E-05	○
Psme1	41.09	75.67	1.84	5.00E-05	142.14	113.18	0.80	1.60E-02	○
Pstpip1	1.84	4.62	2.51	5.00E-05	7.85	4.77	0.61	1.00E-03	○
Ptafr	2.11	5.38	2.55	5.00E-05	9.89	7.07	0.72	3.40E-03	○
Ptgs1	2.99	4.93	1.65	5.00E-05	8.36	6.10	0.73	1.12E-02	○
Pthlh	0.37	3.77	10.23	1.50E-04	4.66	2.82	0.60	2.60E-02	○
Ptpn18	2.14	5.26	2.46	5.00E-05	12.76	7.39	0.58	1.50E-04	
Ptpn5	6.00	52.72	8.78	5.00E-05	50.73	41.89	0.83	3.78E-02	
Ptpn6	2.43	9.61	3.96	5.00E-05	22.68	13.85	0.61	5.00E-05	○
Ptpn7	0.28	2.11	7.62	5.00E-05	4.75	2.37	0.50	3.00E-04	○
Ptprc	0.96	5.85	6.12	5.00E-05	17.19	7.04	0.41	5.00E-05	○
Ptprcap	0.53	3.60	6.74	5.00E-05	23.84	5.81	0.24	5.00E-05	
Ptpro	5.38	20.02	3.72	5.00E-05	20.31	14.98	0.74	2.10E-03	○
Pycard	3.46	17.40	5.02	5.00E-05	31.74	20.72	0.65	1.95E-03	○
Rac2	2.35	16.87	7.18	5.00E-05	45.81	19.49	0.43	5.00E-05	○
Rassf2	3.31	6.30	1.90	5.00E-05	12.40	9.86	0.80	2.29E-02	
Rftn1	1.65	2.61	1.59	1.55E-03	6.22	3.92	0.63	8.00E-04	○
RGD1309808	0.62	1.51	2.44	3.70E-03	10.53	3.28	0.31	5.00E-05	
Rgs1	0.73	4.45	6.10	5.00E-05	11.78	6.03	0.51	5.00E-05	○
Rhoh	0.31	1.16	3.76	2.00E-04	8.44	5.65	0.67	1.42E-02	○
Rin3	3.35	6.00	1.79	5.00E-05	9.08	7.10	0.78	2.93E-02	○
Ripk3	0.99	5.21	5.25	5.00E-05	11.48	8.68	0.76	3.13E-02	○
RT1-Ba	24.06	257.72	10.71	5.00E-05	524.30	231.02	0.44	5.00E-05	
RT1-Bb	18.24	221.39	12.14	5.00E-05	510.76	204.69	0.40	5.00E-05	
RT1-CE10	10.24	21.71	2.12	5.00E-05	44.44	29.31	0.66	2.85E-03	
RT1-Da	28.68	341.17	11.89	5.00E-05	633.98	297.60	0.47	5.00E-05	
RT1-DOa	0.65	9.14	14.12	5.00E-05	18.46	7.08	0.38	5.00E-05	
RT1-DOb	1.01	9.34	9.26	5.00E-05	14.44	6.09	0.42	5.00E-05	
Samsn1	0.23	1.77	7.61	5.50E-04	4.85	2.43	0.50	5.20E-03	○
Sash3	0.57	3.29	5.76	5.00E-05	7.77	3.96	0.51	5.00E-05	○
Sdc1	2.35	34.29	14.61	5.00E-05	36.42	30.33	0.83	4.88E-02	○
Sdf2l1	10.65	17.45	1.64	5.00E-05	29.04	20.52	0.71	3.50E-03	
Sectm1a	0.11	1.08	9.84	4.00E-04	3.53	1.59	0.45	2.00E-04	
Sectm1b	0.25	1.33	5.39	5.00E-04	4.10	2.63	0.64	4.20E-02	
Selplg	1.61	10.18	6.32	5.00E-05	22.65	13.52	0.60	5.00E-05	○
Sema4a	2.96	9.11	3.07	5.00E-05	18.57	11.35	0.61	5.00E-05	○
Serp1	20.55	30.87	1.50	5.00E-05	38.38	29.56	0.77	1.63E-02	
Serpinb6b	0.51	1.95	3.83	5.00E-05	5.79	1.62	0.28	5.00E-05	
Serpine1	1.73	8.72	5.05	5.00E-05	11.03	8.50	0.77	2.00E-02	○
Sh3bp1	1.82	4.97	2.74	5.00E-05	11.98	7.53	0.63	5.00E-05	
Shisa3	5.15	11.30	2.20	5.00E-05	25.11	18.48	0.74	6.15E-03	
Skap2	6.36	19.97	3.14	5.00E-05	28.01	21.12	0.75	7.85E-03	
Sla	0.45	3.08	6.89	5.00E-05	7.02	3.77	0.54	5.00E-05	○
Slamf7	0.39	1.58	4.07	5.00E-05	5.08	2.16	0.42	5.00E-05	○
Slamf8	0.58	2.24	3.87	5.00E-05	6.03	3.30	0.55	3.50E-04	○
Slc11a1	0.70	2.31	3.30	5.00E-05	7.14	3.81	0.53	5.00E-05	○

Slc13a4	6.32	10.47	1.66	5.00E-05	28.65	20.96	0.73	1.50E-03	
Slc14a1	2.26	5.47	2.42	5.00E-05	7.73	2.57	0.33	5.00E-05	
Slc1a1	2.70	5.83	2.16	5.00E-05	6.32	4.70	0.74	4.42E-02	○
Slc1a5	1.43	5.60	3.91	5.00E-05	10.03	7.57	0.75	1.85E-02	○
Slc30a3	0.23	2.00	8.57	5.00E-05	2.32	0.83	0.36	5.00E-05	
Slc38a3	0.67	1.02	1.51	2.83E-02	3.21	2.08	0.65	1.80E-02	
Slc47a1	1.06	2.75	2.58	5.00E-05	8.39	5.79	0.69	4.15E-03	
Slc6a1	2.33	4.07	1.75	5.00E-05	8.32	6.37	0.77	2.54E-02	○
Slfn2	4.13	18.50	4.49	5.00E-05	34.77	23.00	0.66	5.00E-05	○
Smco4	2.08	4.77	2.29	5.00E-05	10.44	7.10	0.68	2.24E-02	
Smoc2	9.65	28.62	2.96	5.00E-05	69.54	49.70	0.71	2.50E-04	
Snx20	1.53	7.04	4.61	5.00E-05	11.29	7.07	0.63	2.35E-03	
Sp140	3.55	8.86	2.50	5.00E-05	16.89	13.41	0.79	2.64E-02	
Spi1	2.02	11.21	5.54	5.00E-05	22.57	15.54	0.69	1.50E-03	○
Spint2	18.54	32.09	1.73	5.00E-05	48.35	36.57	0.76	5.70E-03	○
Spn	0.28	1.29	4.60	5.00E-05	4.29	1.58	0.37	5.00E-05	○
Spns3	0.11	1.26	11.47	1.15E-03	1.89	0.64	0.34	1.00E-04	
Sprr1a	0.67	47.37	70.29	5.00E-05	56.84	41.24	0.73	1.78E-02	○
Spry1	4.54	7.00	1.54	5.00E-05	13.48	10.54	0.78	2.37E-02	
Srgn	5.22	8.70	1.67	5.00E-05	18.96	11.63	0.61	2.50E-04	○
Sstr3	0.17	2.53	15.32	5.00E-05	3.55	1.06	0.30	5.00E-05	
St3gal1	4.29	6.81	1.59	5.00E-05	9.76	7.90	0.81	3.07E-02	
St3gal6	0.62	20.98	33.95	5.00E-05	21.10	16.08	0.76	4.04E-02	○
Stat4	0.28	2.81	10.14	5.00E-05	3.32	1.81	0.55	2.45E-03	○
Stk17b	1.59	3.71	2.34	5.00E-05	7.78	4.69	0.60	3.00E-04	○
Stra6	1.96	8.33	4.25	5.00E-05	27.86	16.61	0.60	5.00E-05	○
Sulf2	14.41	22.24	1.54	5.00E-05	49.13	40.80	0.83	4.28E-02	
Susd3	1.17	2.12	1.81	3.15E-03	3.43	2.29	0.67	4.23E-02	
Syk	0.69	2.78	4.02	5.00E-05	6.57	4.31	0.66	4.50E-04	○
Tagap	0.47	1.26	2.69	5.00E-05	4.41	2.30	0.52	5.00E-05	○
Tapbpl	10.51	15.90	1.51	5.00E-05	27.51	19.32	0.70	6.00E-04	
Tbc1d10c	0.60	2.20	3.65	5.00E-05	8.84	2.83	0.32	5.00E-05	○
Tbxas1	0.98	4.84	4.92	5.00E-05	9.76	7.29	0.75	3.41E-02	○
Tec	4.02	7.61	1.89	5.00E-05	17.64	13.72	0.78	1.62E-02	○
Tes	20.54	33.42	1.63	5.00E-05	36.64	29.59	0.81	2.09E-02	
Tgfbr1	3.26	7.48	2.29	5.00E-05	9.35	7.57	0.81	3.15E-02	○
Tgm2	8.67	14.34	1.65	5.00E-05	32.62	21.60	0.66	5.00E-05	○
Thbd	6.54	31.66	4.84	5.00E-05	36.07	29.11	0.81	1.77E-02	○
Tlr2	0.69	1.88	2.73	5.00E-05	4.17	2.85	0.68	1.54E-02	○
Tmc8	0.99	1.53	1.53	2.32E-02	3.29	1.68	0.51	1.05E-03	
Tmem123	5.36	12.03	2.24	5.00E-05	20.00	13.40	0.67	5.50E-04	
Tnfaip2	1.48	4.35	2.93	5.00E-05	6.80	5.17	0.76	2.31E-02	
Tnfaip8	1.82	5.37	2.95	5.00E-05	9.96	6.76	0.68	4.15E-03	○
Tnfrsf14	0.27	2.18	7.95	5.00E-05	19.33	2.53	0.13	5.00E-05	○
Tnfrsf1b	1.01	2.95	2.93	5.00E-05	7.61	5.12	0.67	1.20E-03	○
Traf1	0.95	1.90	2.00	1.50E-04	6.78	3.28	0.48	5.00E-05	○
Traf3ip3	0.34	1.56	4.55	1.94E-02	6.19	2.31	0.37	1.00E-04	
Trim5	5.73	11.11	1.94	5.00E-05	19.46	14.80	0.76	9.90E-03	○
Tspan13	82.49	176.98	2.15	5.00E-05	162.23	134.71	0.83	4.19E-02	
Ttc7a	2.66	6.69	2.51	5.00E-05	11.20	9.07	0.81	4.46E-02	
Tyrobp	10.98	63.84	5.81	5.00E-05	112.64	87.17	0.77	1.47E-02	○
Ubd	1.02	2.10	2.06	8.40E-03	94.42	4.19	0.04	5.00E-05	○
Unc93b1	8.81	18.56	2.11	5.00E-05	32.86	25.20	0.77	6.45E-03	○
Vav1	0.69	3.22	4.70	5.00E-05	6.79	3.92	0.58	1.00E-04	○
Vcan	0.88	3.45	3.92	5.00E-05	3.09	2.21	0.71	1.01E-02	○
Was	1.20	4.55	3.79	5.00E-05	10.05	6.30	0.63	1.45E-03	○
Xcl1	0.57	19.75	34.85	5.00E-05	6.81	3.23	0.47	2.76E-02	○
Xirp2	0.29	1.21	4.22	5.00E-05	1.96	1.09	0.56	4.25E-03	
Zbp1	0.36	1.69	4.72	1.95E-02	5.49	3.23	0.59	2.30E-03	○
Zc3h12a	1.98	4.03	2.04	5.00E-05	7.36	5.69	0.77	4.48E-02	○
Zc3h12d	0.27	1.11	4.19	5.00E-05	3.33	1.11	0.33	5.00E-05	○
Zdhhc21	10.58	30.40	2.87	5.00E-05	30.98	23.09	0.75	8.50E-04	
Zic2	1.95	3.93	2.02	5.00E-05	13.64	10.56	0.77	2.02E-02	

Supplementary Table 5. List of 164 genes downregulated by SNL and upregulated by Neat1 knockdown (downregulated through Neat1).

Gene	FPKM		Fold change	p_value	FPKM		Fold change	p_value
	CNT	SNL			SNL+Control	SNL+Neat1 shRNA		
Aass	1.20	0.78	0.65	8.55E-03	0.54	0.91	1.69	1.44E-02
Abhd3	18.38	12.06	0.66	5.00E-05	10.57	13.33	1.26	4.93E-02
Abhd8	53.44	27.33	0.51	5.00E-05	21.23	27.18	1.28	1.40E-02
Ache	171.48	31.39	0.18	5.00E-05	41.37	61.03	1.48	2.00E-04
Acsbg1	47.27	25.40	0.54	5.00E-05	21.19	26.20	1.24	2.71E-02
Actg2	2.18	0.92	0.42	5.00E-05	2.30	4.92	2.14	3.00E-04
Adam11	73.49	21.20	0.29	5.00E-05	18.95	25.56	1.35	1.05E-03
Adap1	77.79	44.88	0.58	5.00E-05	37.60	45.00	1.20	4.79E-02
Ass1	52.99	3.22	0.06	5.00E-05	3.00	4.83	1.61	9.45E-03
Atp2b3	21.73	2.81	0.13	5.00E-05	2.54	3.67	1.45	2.05E-03
Atp6v1a	79.69	38.34	0.48	5.00E-05	29.46	35.86	1.22	3.00E-02
B3galt5	3.36	1.54	0.46	5.00E-05	0.98	1.48	1.51	1.38E-02
B3gat2	7.96	0.53	0.07	5.00E-05	0.49	0.97	1.99	1.04E-02
Bend6	73.20	33.69	0.46	5.00E-05	25.97	33.21	1.28	3.15E-02
Bex1	61.87	16.36	0.26	5.00E-05	18.56	24.62	1.33	3.44E-02
C1qtnf4	5.11	0.91	0.18	5.00E-05	0.41	0.77	1.87	4.32E-02
Cabp1	159.40	86.66	0.54	5.00E-05	55.56	69.03	1.24	2.79E-02
Camk2a	30.35	6.59	0.22	5.00E-05	4.67	7.17	1.54	4.50E-04
Camk2g	107.21	52.80	0.49	5.00E-05	50.39	61.94	1.23	3.32E-02
Cdh15	34.00	18.47	0.54	5.00E-05	13.23	17.78	1.34	4.20E-03
Cdh3	8.43	0.11	0.01	5.00E-05	0.32	0.66	2.04	5.00E-03
Chchd10	254.22	106.69	0.42	5.00E-05	74.48	95.07	1.28	1.85E-02
Chga	85.54	18.33	0.21	5.00E-05	10.88	18.59	1.71	5.00E-05
Chgb	116.54	51.48	0.44	5.00E-05	41.27	50.03	1.21	3.40E-02
Chrna6	36.02	1.12	0.03	5.00E-05	0.84	2.14	2.54	5.00E-05
Clcn4	28.21	10.61	0.38	5.00E-05	7.32	9.21	1.26	2.65E-02
Cntnap4	3.56	0.39	0.11	5.00E-05	0.34	0.64	1.89	4.05E-03
Col2a1	14.97	7.44	0.50	5.00E-05	3.46	6.64	1.92	2.90E-03
Cplx2	47.22	27.33	0.58	5.00E-05	31.55	37.89	1.20	4.52E-02
Diras2	17.06	4.83	0.28	5.00E-05	3.32	5.05	1.52	1.15E-02
Dlgap3	21.56	3.70	0.17	5.00E-05	3.25	5.21	1.60	5.50E-04
Dmgdh	3.36	0.50	0.15	5.00E-05	0.30	0.81	2.75	3.50E-04
Dnm3	70.28	21.49	0.31	5.00E-05	17.46	22.03	1.26	2.44E-02
Dpf3	1.60	1.00	0.62	4.14E-02	0.72	1.28	1.77	2.64E-02
Dpp6	93.94	14.33	0.15	5.00E-05	11.64	14.77	1.27	1.75E-02
Dusp15	45.40	25.53	0.56	5.00E-05	17.81	23.37	1.31	4.30E-03
Ephb1	5.17	2.45	0.47	5.00E-05	1.87	2.77	1.48	1.51E-02
Ephx1	29.06	18.01	0.62	5.00E-05	24.06	29.66	1.23	3.67E-02
Fads6	21.54	3.89	0.18	5.00E-05	2.49	3.53	1.42	2.75E-02
Faim2	84.86	11.82	0.14	5.00E-05	4.32	8.18	1.89	1.00E-04
Fbln5	54.15	31.42	0.58	5.00E-05	26.77	34.52	1.29	7.55E-03
Flrt1	1.71	0.21	0.12	5.00E-05	0.16	0.47	3.00	1.13E-02
Fn3k	4.24	1.78	0.42	5.00E-05	1.69	3.02	1.78	2.80E-02
Frmd3	13.55	6.22	0.46	5.00E-05	4.60	7.18	1.56	8.50E-04
Gabra1	13.89	1.30	0.09	5.00E-05	0.60	1.03	1.74	7.90E-03
Gabrg2	50.42	6.57	0.13	5.00E-05	3.44	5.39	1.57	1.25E-02
Galnt5	9.73	0.58	0.06	5.00E-05	0.52	0.88	1.69	1.60E-02
Gfra4	1.29	0.74	0.57	8.60E-03	0.70	1.12	1.61	4.34E-02
Gnai1	37.09	23.05	0.62	5.00E-05	20.10	25.80	1.28	1.40E-02
Gng7	7.97	3.87	0.49	5.00E-05	2.11	2.98	1.41	3.29E-02
Gpr27	3.90	2.14	0.55	5.00E-05	1.97	2.89	1.47	2.47E-02
Gramd1b	55.05	34.56	0.63	5.00E-05	31.33	39.63	1.26	2.07E-02
Grem2	16.45	1.72	0.10	5.00E-05	1.41	2.59	1.84	6.50E-04
Grin1	32.35	10.78	0.33	5.00E-05	9.54	11.72	1.23	4.29E-02
Gsta1	34.14	22.06	0.65	5.00E-05	24.65	33.97	1.38	3.05E-03
Gstm7	62.28	19.33	0.31	5.00E-05	15.20	20.10	1.32	2.32E-02
Hhatl	26.36	4.60	0.17	5.00E-05	2.55	3.68	1.44	4.33E-02
Htr3a	66.67	0.52	0.01	5.00E-05	0.20	0.89	4.37	1.50E-04
Htr3b	68.49	0.61	0.01	5.00E-05	0.15	0.60	4.06	3.50E-04
Htr4	5.46	2.60	0.48	5.00E-05	2.28	3.16	1.39	2.90E-02
Igfbp2	34.01	15.11	0.44	5.00E-05	53.44	69.49	1.30	1.78E-02
Il31ra	13.81	0.31	0.02	5.00E-05	0.38	1.42	3.77	5.00E-05
Inpp5j	14.68	5.25	0.36	5.00E-05	3.41	4.52	1.33	4.38E-02
Iqsec3	7.87	4.83	0.61	5.00E-05	3.78	4.99	1.32	9.30E-03
Kank4	19.18	11.90	0.62	5.00E-05	12.51	15.31	1.22	3.91E-02

Kazn	18.31	9.42	0.51	5.00E-05	7.67	9.60	1.25	3.52E-02
Kbtbd11	11.35	6.61	0.58	5.00E-05	4.98	7.17	1.44	7.00E-04
Kcna2	61.93	12.68	0.20	5.00E-05	9.50	14.30	1.50	5.00E-05
Kcnab2	231.44	60.22	0.26	5.00E-05	41.24	52.52	1.27	1.51E-02
Kcnc3	26.81	3.41	0.13	5.00E-05	2.29	3.03	1.32	4.00E-02
Kcnh2	42.47	17.94	0.42	5.00E-05	12.11	15.31	1.26	1.92E-02
Kcnh6	9.84	0.99	0.10	5.00E-05	0.91	1.67	1.83	1.55E-03
Kcnk1	26.39	11.07	0.42	5.00E-05	7.52	10.52	1.40	5.40E-03
Lamp5	18.96	0.73	0.04	5.00E-05	1.27	2.37	1.87	1.35E-03
Lcn2	5.75	3.01	0.52	1.50E-04	1.08	1.96	1.81	2.90E-02
Lgi3	108.43	20.98	0.19	5.00E-05	11.63	18.05	1.55	5.00E-05
Lix1	5.38	0.40	0.08	5.00E-05	0.24	0.66	2.79	6.95E-03
LOC691995	27.39	9.58	0.35	5.00E-05	8.98	12.47	1.39	1.31E-02
Map7	26.96	10.64	0.39	5.00E-05	6.94	9.04	1.30	1.39E-02
Mccc2	10.14	6.18	0.61	5.00E-05	7.01	9.13	1.30	3.92E-02
Mgll	80.31	38.24	0.48	5.00E-05	25.65	33.61	1.31	6.25E-03
Mrap	26.31	14.79	0.56	5.00E-05	11.30	15.28	1.35	4.88E-02
Mrgprb5	0.85	0.52	0.61	2.68E-01	0.04	0.40	10.69	1.00E+00
Mst1r	3.38	0.96	0.29	5.00E-05	0.84	1.37	1.62	4.25E-03
Mt3	710.51	68.74	0.10	5.00E-05	40.66	80.99	1.99	5.00E-05
Nat8l	144.24	31.91	0.22	5.00E-05	18.33	26.86	1.47	1.00E-04
Ncald	56.64	27.18	0.48	5.00E-05	19.85	26.34	1.33	2.95E-03
Necab3	75.96	23.65	0.31	5.00E-05	13.79	17.62	1.28	3.50E-02
Nmb	92.44	3.09	0.03	5.00E-05	2.54	4.47	1.76	1.51E-02
Nppb	3.11	0.18	0.06	4.90E-03	0.33	1.09	3.30	2.34E-02
Nptx1	60.75	1.73	0.03	5.00E-05	1.51	3.06	2.03	5.00E-05
Nrarp	12.90	6.50	0.50	5.00E-05	5.43	7.39	1.36	1.93E-02
Nrn1	221.01	8.24	0.04	5.00E-05	8.17	12.59	1.54	5.30E-03
Olfm1	168.30	80.56	0.48	5.00E-05	63.16	78.44	1.24	3.74E-02
Pacsin1	74.74	13.35	0.18	5.00E-05	11.21	14.74	1.31	1.41E-02
Panx2	47.00	14.69	0.31	5.00E-05	8.41	15.18	1.81	5.00E-05
Parm1	127.30	13.08	0.10	5.00E-05	9.15	14.33	1.57	9.00E-04
Pcmt1	104.47	58.78	0.56	5.00E-05	37.68	48.23	1.28	9.15E-03
Pcp4l1	19.97	3.93	0.20	5.00E-05	3.28	5.19	1.58	6.95E-03
Pde4c	5.81	2.26	0.39	5.00E-05	2.15	3.06	1.42	3.15E-02
Pdlim3	1.54	0.54	0.35	1.50E-04	0.32	1.10	3.41	5.50E-04
Pdxp	19.44	10.73	0.55	5.00E-05	9.82	12.55	1.28	3.51E-02
Peg3	61.20	32.49	0.53	5.00E-05	29.11	35.17	1.21	3.71E-02
Pgf	6.28	2.92	0.46	5.00E-05	2.60	3.94	1.52	2.49E-02
Phgdh	56.80	37.24	0.66	5.00E-05	32.15	40.75	1.27	1.53E-02
Pianp	17.87	3.22	0.18	5.00E-05	3.40	4.85	1.43	2.25E-02
Pkp2	1.10	0.71	0.65	2.44E-02	0.41	0.86	2.09	4.45E-03
Pla2g3	14.06	3.62	0.26	5.00E-05	2.45	4.12	1.68	2.00E-03
Plekhd1	53.04	6.62	0.12	5.00E-05	5.58	8.05	1.44	1.95E-03
Plxna3	13.33	8.31	0.62	5.00E-05	6.01	7.97	1.33	4.60E-03
Pmp2	182.24	86.92	0.48	5.00E-05	30.34	54.66	1.80	5.00E-05
Rab9b	24.49	8.14	0.33	5.00E-05	3.68	7.11	1.93	5.00E-05
Rgs4	377.31	17.24	0.05	5.00E-05	22.55	32.96	1.46	5.00E-05
Rims3	33.18	17.47	0.53	5.00E-05	14.45	17.99	1.25	2.55E-02
Rnf112	37.39	2.76	0.07	5.00E-05	1.67	3.42	2.04	5.00E-05
Rph3a	137.83	31.88	0.23	5.00E-05	19.98	29.93	1.50	5.00E-05
Rtn4rl1	39.25	7.91	0.20	5.00E-05	5.78	7.37	1.27	4.04E-02
Rtn4rl2	18.77	4.64	0.25	5.00E-05	2.92	4.17	1.43	4.71E-02
Ryr2	7.07	2.91	0.41	5.00E-05	1.43	2.44	1.71	5.00E-05
Samd14	65.80	33.48	0.51	5.00E-05	27.00	36.62	1.36	3.75E-03
Scg2	163.07	7.61	0.05	5.00E-05	9.10	17.71	1.95	5.00E-05
Scn11a	73.02	1.06	0.01	5.00E-05	2.19	4.30	1.97	5.00E-05
Scn1a	12.24	2.49	0.20	5.00E-05	1.06	1.97	1.87	5.00E-05
Scn4b	164.24	84.87	0.52	5.00E-05	54.32	67.83	1.25	1.81E-02
Scn8a	32.40	8.17	0.25	5.00E-05	5.01	7.01	1.40	4.00E-04
Scrg1	11.03	2.50	0.23	5.00E-05	0.88	1.94	2.21	3.32E-02
Sema6b	3.59	2.00	0.56	5.00E-05	1.83	2.68	1.46	1.33E-02
Sfrp5	180.37	104.42	0.58	5.00E-05	69.30	89.81	1.30	3.50E-03
Sgsm1	18.37	8.68	0.47	5.00E-05	7.21	9.19	1.27	1.77E-02
Sh3gl2	60.26	17.73	0.29	5.00E-05	13.65	17.97	1.32	2.07E-02
Slc22a17	128.60	73.04	0.57	5.00E-05	67.37	83.67	1.24	1.68E-02
Slit1	27.70	13.02	0.47	5.00E-05	9.69	12.32	1.27	1.08E-02
Smad9	16.58	1.63	0.10	5.00E-05	0.96	1.83	1.91	7.50E-04
Sparcl1	107.61	71.07	0.66	5.00E-05	90.63	122.39	1.35	1.95E-03
Spire2	46.81	18.16	0.39	5.00E-05	12.36	17.35	1.40	1.35E-03

Sst	159.65	0.59	0.00	5.00E-05	0.93	1.99	2.15	1.71E-02
Susd4	83.94	21.07	0.25	5.00E-05	14.22	18.46	1.30	8.75E-03
Sv2c	143.99	67.25	0.47	5.00E-05	38.59	58.27	1.51	5.00E-05
Svip	79.11	43.27	0.55	5.00E-05	31.78	44.15	1.39	2.40E-03
Syt5	26.02	13.07	0.50	5.00E-05	12.54	17.38	1.39	5.40E-03
Syt6	11.22	1.22	0.11	5.00E-05	1.35	2.09	1.54	2.49E-02
Syt9	33.68	2.75	0.08	5.00E-05	2.68	4.22	1.57	4.70E-03
Tac1	248.18	46.82	0.19	5.00E-05	30.18	43.57	1.44	1.10E-03
Thsd7a	12.32	5.68	0.46	5.00E-05	5.29	6.62	1.25	4.05E-02
Tll1	4.14	0.92	0.22	5.00E-05	0.83	1.38	1.66	4.30E-03
Tmem151b	14.37	0.40	0.03	5.00E-05	0.68	1.15	1.69	1.42E-02
Tmem179	65.68	41.88	0.64	5.00E-05	32.68	39.45	1.21	4.27E-02
Tmem229a	15.12	9.20	0.61	5.00E-05	7.57	10.58	1.40	1.10E-03
Tmem25	25.34	4.35	0.17	5.00E-05	3.69	5.05	1.37	3.33E-02
Tmem255a	38.08	1.58	0.04	5.00E-05	0.82	1.42	1.73	9.10E-03
Tmem45b	12.77	2.26	0.18	5.00E-05	2.33	3.31	1.42	2.09E-02
Tomm34	43.35	24.16	0.56	5.00E-05	20.24	25.64	1.27	2.21E-02
Tox2	20.61	3.24	0.16	5.00E-05	2.42	3.61	1.50	2.00E-02
Trpa1	24.72	2.72	0.11	5.00E-05	0.31	0.50	1.60	5.64E-01
Trpm8	27.84	6.22	0.22	5.00E-05	5.28	6.68	1.27	4.18E-02
Tspyl4	88.09	28.33	0.32	5.00E-05	21.30	26.58	1.25	2.42E-02
Tst	20.41	9.50	0.47	5.00E-05	7.55	11.00	1.46	1.44E-02
Tyrp1	117.79	27.18	0.23	5.00E-05	14.28	17.99	1.26	2.65E-02
Unc5a	2.47	1.15	0.46	5.00E-05	0.78	1.39	1.78	5.55E-03
Vsnl1	104.88	7.30	0.07	5.00E-05	3.82	7.15	1.87	1.50E-04
Vwa5b2	22.54	5.67	0.25	5.00E-05	4.16	6.00	1.44	9.05E-03
Wnk2	9.90	5.46	0.55	5.00E-05	3.78	4.87	1.29	1.90E-02
Zdhhc19	2.71	1.03	0.38	5.00E-05	0.64	1.04	1.61	4.01E-02
Zfp483	2.58	1.53	0.59	2.50E-04	1.61	2.38	1.48	3.91E-02

Supplementary Table 6. List of Neat1-interacting genes predicted by RIblast.
A calculated interaction energy (kcal/mol) of Neat1 to each gene was shown.
Genes associated with the inflammatory response predicted by IPA were also indicated.

Genes upregulated through Neat1			Genes downregulated through Neat1	
106 genes			76 genes	
Gene	Energy (kcal/mol)	Inflammatory response	Gene	Energy (kcal/mol)
Cd244	-30863.78	○	Kcnc3	-45638.47
Xcl1	-27340.97	○	Wnk2	-27262.29
Igf2	-25413.91	○	Flrt1	-27142.25
Mir205	-24834.49		Tmem229a	-26795.66
Shisa3	-23696.83		Tmem151b	-26785.69
Sstr3	-23516.89		Htr4	-26450.89
Adgrg5	-23045.38		Iqsec3	-26355.68
Xirp2	-20184.18		Kcna2	-25396.71
Ptgs1	-20113.15	○	Sgsm1	-25112.06
Hcn4	-19574.81		Faim2	-23154.06
St3gal6	-19124.26	○	Camk2a	-22773.65
Apobr	-19004.25		Scn8a	-21953.17
Arhgap4	-18711.61		Scn4b	-21827.83
Myrf	-18623.02	○	Nptx1	-21769.37
Foxp4	-18313.93		Cplx2	-21373.58
Hr	-17903.60		Ryr2	-20587.77
Elf4	-17340.30	○	Il31ra	-19184.03
Flnc	-16926.61	○	Frmd3	-18882.80
Prss12	-16579.00		Pianp	-17809.37
Vcan	-16284.28	○	Sv2c	-17574.16
Mki67	-16043.78	○	Slit1	-17402.07
St3gal1	-15669.07		Nat8l	-16921.34
Akna	-15660.33	○	Cntnap4	-16582.14
Psd4	-15656.19		Gramd1b	-16559.58
Ddr2	-14738.15	○	Thsd7a	-16507.67
Gipc3	-14639.83		Map7	-16159.69
Fbn2	-14428.21		Rims3	-15949.03
Fndc1	-14176.44		Atp2b3	-15253.43
Pou2f2	-13704.68	○	Dpf3	-15220.55
Lif	-13693.24	○	Galnt5	-14725.03
Parvg	-13678.66		Plxna3	-14290.80
Selplg	-13602.86	○	Peg3	-14257.94
Mir146b	-13340.66		Pacsin1	-13455.00
Sp140	-13287.13		Parm1	-13267.28
Csf2rb	-12929.47	○	Ephb1	-13202.80
Adap2	-12799.30		Vwa5b2	-12986.38
Tnfrsf1b	-12648.52	○	Kbtbd11	-12904.06
Pycard	-12633.85	○	Cdh3	-12885.09
Pik3r5	-12615.76	○	Sema6b	-12672.17
Sh3bp1	-12518.24		Diras2	-12505.69
Dhfr	-12269.73	○	Rnf112	-12101.48
Serpine1	-12267.62	○	Fads6	-11958.51
Nlrp1a	-12179.76	○	Ache	-11894.00
Slamf7	-12079.42	○	Dnm3	-11673.61
Ciita	-12071.38	○	Dlgap3	-11666.65
Dock8	-12015.02	○	Chgb	-11628.29
Slc11a1	-11919.55	○	Mst1r	-11404.06
Dok2	-11900.95	○	Unc5a	-11251.00
Arhgap30	-11897.90		Gng7	-10829.78
Dennd1c	-11504.71		Tox2	-10803.70

Rin3	-11328.24	○	Col2a1	-10599.48	
Adamts12	-11284.62	○	Inpp5j	-10548.96	
Cd300lf	-11182.72	○	Tspyl4	-10540.37	
Ttc7a	-11158.03		Lgi3	-10220.15	
Zc3h12d	-11008.94	○	Pde4c	-10093.05	
Ikbke	-10966.55	○	Mgll	-10016.36	
Gpr31	-10931.77	○	Scn1a	-9882.79	
Zdhhc21	-10901.29		Kcnab2	-9734.38	
Cd80	-10877.32	○	Kazn	-9658.22	
Cd4	-10720.46	○	Kank4	-9394.87	
Bcl3	-10678.44	○	Zfp483	-9373.92	
Hk2	-10647.89		Adam11	-9352.44	
Lrrc18	-10647.53		Scn11a	-9176.89	
Sema4a	-10645.50	○	Plekhd1	-9132.94	
Fgd3	-10590.51		Camk2g	-9093.35	
Dab2	-10585.67		Dusp15	-8716.49	
Cd93	-10583.27	○	Grin1	-8694.54	
Ikzf1	-10516.69		Chga	-8675.30	
Pde7a	-10503.98	○	Tmem45b	-8655.06	
Syk	-10217.34	○	Smad9	-8609.93	
Adcyap1	-10089.11	○	Trpm8	-8560.25	
Arhgap11a	-10050.32		Kcnh6	-8517.32	
Itgb8	-10043.84	○	Clcn4	-8359.98	
Irf5	-10004.79	○	Rph3a	-8213.32	
Asb2	-9739.49	○	Adap1	-8112.47	
Zc3h12a	-9712.12	○	Slc22a17	-8019.06	
Ptpn5	-9676.07				
C3	-9593.35	○			
Rassf2	-9361.88				
Ets1	-9190.19	○			
Nckap1l	-9188.26	○			
Csf2ra	-9177.05	○			
Cd37	-9145.69	○			
Prr5l	-9137.87				
Tnfrsf14	-9127.49	○			
Itln1	-9124.49	○			
Emp1	-9081.87				
Fem1c	-9066.42				
Spn	-8957.95	○			
Stra6	-8879.81	○			
Sulf2	-8844.70				
Npas2	-8832.24				
Cyp1b1	-8820.50	○			
Slc6a1	-8806.22	○			
Tmc8	-8737.31				
Galntl6	-8657.96				
Ptafr	-8634.34	○			
Nlrp3	-8619.09	○			
Cd22	-8542.03	○			
Parp14	-8462.77				
Maff	-8389.39				
Cd101	-8376.16				
Il1rn	-8303.18	○			
Pdgfra	-8158.70	○			
Cpne8	-8117.16				
Gpr4	-8055.93	○			

Supplementary Table 7. List of non-interacting genes with Neat1 predicted by RIblast.
A calculated interaction energy (kcal/mol) of Neat1 to each gene was shown.
Genes associated with the immunological disease were also indicated.

Genes upregulated through Neat1			Genes downregulated through Neat1	
283 genes			88 genes	
Gene Symbol	Energy	Immunological disease	Gene Symbol	Energy
Slfn2	-7942.18	○	Cdh15	-7992.90
Il4r	-7916.00	○	Syt9	-7969.19
Pik3ap1	-7876.07	○	Tll1	-7954.35
Il17ra	-7835.35	○	Kcnh2	-7556.57
Itgal	-7823.46	○	Susd4	-7479.26
Edn3	-7761.87	○	B3galt5	-7421.31
RT1-DOb	-7704.82		Syt6	-7380.48
Kcnj15	-7702.73	○	Tmem255a	-6984.13
Ptprc	-7615.82	○	Rtn4rl2	-6967.27
Cdkn1a	-7613.47	○	Sfrp5	-6953.17
Artn	-7580.70		Panx2	-6891.20
Mycl	-7564.57	○	Trpa1	-6818.93
Acap1	-7536.00		Dpp6	-6799.56
Lcp2	-7510.39	○	Hhatl	-6529.21
Zic2	-7500.03		Rtn4rl1	-6460.32
Tnfaip2	-7477.46		Olfm1	-6423.81
RT1-CE10	-7454.20		Sh3gl2	-6402.12
Cyth4	-7437.14		Syt5	-6345.80
Baz1a	-7436.18		Grem2	-6342.44
Il10ra	-7345.89	○	Pla2g3	-6034.27
Mefv	-7339.54	○	Chrna6	-5951.59
Fgr	-7286.30	○	Zdhhc19	-5820.39
Pi15	-7228.07		Spire2	-5598.20
RT1-Bb	-7222.67		Tmem25	-5590.78
Limd2	-7197.07		Pcp4l1	-5504.85
Csrnp1	-7142.81		Svip	-5498.26
Tgfbr1	-7077.78	○	Gpr27	-5274.90
Anxa3	-7051.78	○	Atp6v1a	-5144.35
Mpeg1	-6985.32	○	Ass1	-5124.00
Il2rg	-6975.68	○	Rab9b	-4917.99
Nfkb2	-6971.52	○	Ncald	-4865.83
Nck2	-6951.83		Gabra1	-4824.19
Abcb4	-6887.73	○	Scrg1	-4754.60
Apbb1ip	-6833.79	○	Htr3b	-4744.70
Ocln	-6830.29	○	Tomm34	-4686.39
Ly6c	-6823.64		Nrarp	-4671.37
Pld4	-6805.43	○	Phgdh	-4669.67
Met	-6782.15	○	Scg2	-4655.54
Hcls1	-6763.68	○	Sparcl1	-4608.73
Clec2g	-6757.90		Tmem179	-4572.98
Myo1f	-6753.56	○	Gabrg2	-4548.57
Vav1	-6718.68	○	B3gat2	-4523.31
Jak2	-6711.83	○	Pkp2	-4458.99
Slc13a4	-6633.56		Samd14	-4345.40
Cybb	-6633.20	○	Lamp5	-4256.01
Efhd2	-6592.89		Fbln5	-4170.10
Ptpn7	-6576.82		Gfra4	-4105.64
Itgae	-6573.20	○	Pcmt1	-3919.15
Tgm2	-6568.60	○	Pdxp	-3900.27
Igtp	-6522.91		Rgs4	-3861.83
Ccl22	-6496.26	○	Fn3k	-3813.31
Thbd	-6440.76	○	Gstm7	-3743.22

Aldh1a3	-6434.40		Acsbg1	-3727.32
C2	-6433.22	○	Dmgdh	-3699.64
Slc14a1	-6310.04		Kcnk1	-3691.35
Ptpro	-6244.05	○	Htr3a	-3621.07
Clec2d2	-6228.80		Tyrp1	-3579.45
Slc30a3	-6228.48		Gnai1	-3504.47
Mob1a	-6191.11		Pgf	-3401.85
Lox	-6142.35		Abhd8	-3295.88
Igfbp3	-6098.79	○	Lix1	-3255.62
Mdfic	-6072.98		Necab3	-3125.69
Cxcl9	-6043.62	○	Aass	-2991.82
Dapp1	-6013.11		Igfbp2	-2927.20
Tmem123	-5971.69		Chchd10	-2889.49
Ripk3	-5966.14	○	Mccc2	-2861.40
Eya2	-5957.54	○	Cabp1	-2791.68
Lcp1	-5919.71	○	Tst	-2731.70
Osr1	-5919.56		Bend6	-2730.62
Map4k1	-5918.29	○	Pmp2	-2696.45
Lfng	-5905.63	○	Nrn1	-2634.26
Foxc1	-5821.89		Ephx1	-2582.24
Slc1a1	-5791.63		Vsnl1	-2546.30
Mapkapk2	-5783.66	○	Lcn2	-2417.43
Lyn	-5774.81	○	Mrgprb4	-2408.60
Fes	-5744.21	○	Actg2	-2383.00
Irf8	-5714.37	○	C1qtnf4	-2344.53
Il21r	-5692.37	○	Abhd3	-2279.29
Arhgap9	-5682.82		Sst	-2254.33
Laptm5	-5645.56	○	Bex1	-2163.76
Cysltr1	-5642.43	○	Mrap	-2132.63
Pstpip1	-5614.32	○	Pdlim3	-1962.84
Klra1	-5555.74		LOC691995	-1940.66
LOC308990	-5552.15		Nmb	-1805.47
Slc1a5	-5547.97		Mt3	-1482.12
Klf6	-5540.49	○	Nppb	-1458.81
Cldn4	-5428.53		Gsta1	-1377.13
Cotl1	-5400.50		Tac1	-638.79
Ncf1	-5360.30	○		
G2e3	-5351.30			
Cd6	-5346.09	○		
Cd38	-5330.22	○		
Nlrc4	-5294.96	○		
Parp9	-5190.02	○		
Sdc1	-5176.86	○		
C5ar1	-5167.92	○		
Klhl6	-5097.10	○		
Sla	-5087.70	○		
Fgd2	-5084.50			
Arhgap15	-5084.09	○		
Itgb7	-5072.57	○		
Adamts1	-5063.08			
Spry1	-5054.50			
Cldn1	-5050.78			
Ptpn18	-5048.46			
Slamf8	-4988.77	○		
Htra3	-4947.52	○		
Sprr1a	-4915.45			
Clec9a	-4871.55	○		
Nfil3	-4857.64	○		
Slc38a3	-4842.09			

Asgr2	-4807.01			
Col26a1	-4797.84			
C1s	-4796.05	○		
Gna15	-4781.31	○		
Lck	-4751.00	○		
Rac2	-4707.82	○		
Tbc1d10c	-4706.61	○		
Gjb2	-4695.54			
Itgb2	-4665.48	○		
Gsdmd	-4658.41	○		
Il2rb	-4655.66	○		
Ccdc69	-4633.81			
Rftn1	-4618.26	○		
P2ry13	-4611.13	○		
Il13ra1	-4593.96	○		
Cish	-4572.65	○		
Lat2	-4508.04	○		
Ankrd1	-4494.85			
Arsi	-4494.58			
Acp5	-4462.89	○		
Icam1	-4462.84	○		
Tapbpl	-4439.47			
Was	-4411.51	○		
Snx20	-4390.97			
Ccr5	-4382.91	○		
Cldn11	-4281.63	○		
Cdh17	-4263.86	○		
Spi1	-4222.43	○		
Skap2	-4212.11			
Klrk1	-4194.35	○		
Gbp5	-4188.08			
Arrdc4	-4164.27			
Unc93b1	-4129.51	○		
Kcnn4	-4114.23	○		
Slc47a1	-4105.61			
Lag3	-4077.55	○		
Ncf4	-4074.51	○		
Adgre1	-4062.75			
Gbp2	-4042.36	○		
Ptpn6	-4042.19	○		
RGD1309808	-4027.43			
Susd3	-3997.03			
Spns3	-3967.06			
Trim5	-3961.20	○		
Tagap	-3957.07	○		
Lce1f	-3950.16			
Fam111a	-3902.83			
Casp8	-3893.47	○		
Cd74	-3882.23	○		
Tec	-3870.53	○		
Postn	-3863.57	○		
Enpp3	-3832.07	○		
Ifi47	-3761.51	○		
Irgm	-3741.18			
Fcrla	-3740.27			
Cd3e	-3696.07	○		
Irf1	-3693.40	○		
Plat	-3691.67	○		
Btk	-3686.23	○		

Arhgdib	-3666.56	〇		
Smoc2	-3657.41			
Pla2g4a	-3645.40	〇		
Gpr84	-3618.23	〇		
Clec4a1	-3601.00			
Cyp4b1	-3599.09	〇		
Cytip	-3597.37	〇		
Tlr2	-3586.43	〇		
Cdca7	-3536.45	〇		
Gpr171	-3523.71			
Sash3	-3513.02	〇		
Bst2	-3493.64	〇		
B3galt2	-3490.70	〇		
Ncf2	-3472.82	〇		
Serpinb6b	-3409.62			
Rgs1	-3376.73	〇		
Clec12a	-3366.82	〇		
Neurl3	-3293.97			
Lat	-3293.54	〇		
Stk17b	-3267.99	〇		
Cst7	-3211.19			
Sectm1a	-3189.85			
Traf3ip3	-3186.75	〇		
Gimap7	-3174.95			
Msln	-3171.72			
Batf	-3155.69	〇		
Epsti1	-3153.57	〇		
Asgr1	-3124.41			
Stat4	-3124.25	〇		
Fcnb	-3103.53			
Lyc2	-3094.95			
Tbxas1	-3075.52	〇		
Traf1	-3068.76	〇		
Plek	-3044.75	〇		
Nfkbiz	-3013.20	〇		
Lilrb4	-3007.54	〇		
Spint2	-2990.58			
Csrp3	-2956.50			
Map3k8	-2950.68	〇		
Casq2	-2890.09			
Tes	-2880.69			
Ctse	-2872.14			
Nfkbia	-2857.50	〇		
Zbp1	-2800.25	〇		
Apol3	-2777.90			
Fap	-2732.49	〇		
Sectm1b	-2663.87			
Cep55	-2630.43			
Mmd	-2623.30	〇		
Ctsw	-2608.74	〇		
Cd53	-2608.11	〇		
Aurkb	-2598.25	〇		
Fam180a	-2587.14			
Tnfaip8	-2559.64			
Ccl7	-2529.88	〇		
Hmgb2	-2522.29			
Gpr65	-2507.60	〇		
Cxcr5	-2487.15	〇		
Psme1	-2457.70			

RT1-Ba	-2425.02			
Arg1	-2407.40	〇		
Tspan13	-2378.62			
Cmtm2a	-2336.96			
Lyz2	-2332.97			
LOC100910973	-2308.97			
RT1-DOa	-2274.38			
Napsa	-2265.66	〇		
Ctla2a	-2224.44			
Gal	-2203.01	〇		
RT1-Da	-2123.70			
Cxcl13	-2101.78	〇		
Ifitm1	-2007.36			
Il1b	-1924.07	〇		
Cd40	-1858.54	〇		
Ptprcap	-1829.41	〇		
Sdf2l1	-1775.46			
Rhoh	-1773.28	〇		
Clec4a	-1739.64	〇		
Fcgr3a	-1733.48			
Crabp2	-1733.19			
Srgn	-1731.60	〇		
Smco4	-1706.03			
Gzmb	-1681.22	〇		
Ms4a7	-1677.19	〇		
Serp1	-1633.92	〇		
Mnda	-1580.15			
Akr1b8	-1570.04			
Samsn1	-1550.13			
Ism1	-1514.64			
Lst1	-1486.15	〇		
Btg1	-1435.51	〇		
Clec4a3	-1434.66	〇		
Ly86	-1418.94	〇		
Pdcd2	-1413.25			
Tyrobp	-1352.31	〇		
Cd3g	-1315.07	〇		
Ccl2	-1295.03	〇		
Hcst	-1291.64	〇		
Psmb9	-1276.71	〇		
Casp1	-1264.17	〇		
Ubd	-1149.60	〇		
Crisp1	-1071.47			
Gmfg	-1038.52			
Pim1	-1015.34	〇		
Cyba	-1010.56	〇		
Cck	-989.13			
Cd3d	-933.93	〇		
Mir221	-832.77			
Aif1	-821.37	〇		
Plac8	-759.66	〇		
Pthlh	-665.71			
Bcl2a1	-559.93	〇		
Ccl17	-267.50	〇		
